# An in vitro model of neuronal ensembles

**DOI:** 10.1038/s41467-022-31073-1

**Published:** 2022-06-09

**Authors:** M. Angeles Rabadan, Estanislao Daniel De La Cruz, Sneha B. Rao, Yannan Chen, Cheng Gong, Gregg Crabtree, Bin Xu, Sander Markx, Joseph A. Gogos, Rafael Yuste, Raju Tomer

**Affiliations:** 1 Department of Biological Sciences, Columbia University, New York, NY USA; 2 Mortimer B. Zuckerman Mind Brain and Behavior Institute, Columbia University, New York, NY USA; 3 Department of Biomedical Engineering, Columbia University, New York, NY USA; 4 Department of Psychiatry, Vagelos College of Physicians & Surgeons, Columbia University, New York, NY USA; 5 Department of Physiology, Columbia University, New York, NY USA; 6 Department of Neuroscience, Columbia University, New York, NY USA; 7 Department of Psychiatry, Columbia University, New York, NY USA; 8 NeuroTechnology Center, Columbia University, New York, NY USA

**Keywords:** Schizophrenia, Neural circuits, Cellular neuroscience

## Abstract

Advances in 3D neuronal cultures, such as brain spheroids and organoids, are allowing unprecedented in vitro access to some of the molecular, cellular and developmental mechanisms underlying brain diseases. However, their efficacy in recapitulating brain network properties that encode brain function remains limited, thereby precluding development of effective in vitro models of complex brain disorders like schizophrenia. Here, we develop and characterize a Modular Neuronal Network (MoNNet) approach that recapitulates specific features of neuronal ensemble dynamics, segregated local-global network activities and a hierarchical modular organization. We utilized MoNNets for quantitative in vitro modelling of schizophrenia-related network dysfunctions caused by highly penetrant mutations in *SETD1A* and *22q11.2* risk loci. Furthermore, we demonstrate its utility for drug discovery by performing pharmacological rescue of alterations in neuronal ensembles stability and global network synchrony. MoNNets allow in vitro modelling of brain diseases for investigating the underlying neuronal network mechanisms and systematic drug discovery.

## Abstract (web summary)

Advances in 3D neuronal cultures have allowed unprecedented access to the mechanisms underlying brain diseases. This work describes the novel Modular Neuronal Network (MoNNet) system, which enables more complex studies of cortical neuronal ensemble dynamics.

## Introduction

Recent advances in three-dimensional (3D) neuronal cultures are providing effective means for non-invasive modeling of specific neuronal processes in vitro^1–6^. For example, the brain spheroids and organoids have been successfully used to investigate the cell biological and developmental mechanisms underlying autism spectrum disorders (ASD)^7,8^. Despite this progress, the state-of-the-art 3D neuronal cultures still lack complex (i.e. beyond single neuronal patterns) features^9,10^ that are reminiscent of in vivo brain functional networks, limiting their utility for in vitro modeling of brain diseases such as schizophrenia (SCZ) and other neuropsychiatric disorders, where local and global network dysfunction appears to be a common convergence point of diverse etiological factors^11–14^. Recent approaches of increasing the size of 3D neuronal cultures^8,15^ may further increase the captured functional complexity, however, the inaccessibility of nutrients and oxygen supply to the inner cores remains a major technical hurdle. Moreover, the enhanced size may further limit the reagents penetrability and optical accessibility.

We explored an alternate 3D cell-culture approach to mitigate some of these limitations, with an eventual goal to develop quantitative in vitro models of the compromised signal propagation in brain networks associated with SCZ and other neuropsychiatric disorders^12^. Our approach builds upon the facts that the mammalian brain architecture is hierarchically modular with small-world network architecture i.e. highly intra-connected modules (specific brain regions) with fewer inter-modular connections across the system^16^. For example, the dominant successful computational models simulate cortical function by implementing brain networks as highly intra-connected modules of neurons with fewer inter-modular connections^17^. To speedily test and validate the approach by robust comparisons to an extensive in vivo knowledge base, we used dissociated hippocampal cells from late stage mouse embryos to develop self-organized networks of spheroid-like modular units, termed Modular Neuronal Network (MoNNet). We performed exhaustive molecular, cellular and network function characterization of MoNNets by RNAseq experiments, immunostaining, systems-level cellular-resolution Ca^2+^ imaging over several weeks of culture age, and systematic pharmacological interventions. These experiments revealed diverse neuronal activity patterns, segregated local-global network activity, formation and maintenance of stable ensembles/modules and a hierarchical organization of modules with varied strength functional connections.

We then used the MoNNet approach to develop and characterize in vitro quantitative models of network dysfunctions induced by two bona fide SCZ-risk mutations. SCZ is characterized by psychosis, cognitive symptoms as well as negative symptoms such as social and emotional withdrawal. Research in humans and model organisms has identified a diverse set of changes in structural connectivity, synaptic plasticity and excitatory–inhibitory (E–I) balance as well as altered neuromodulation^18,19^ reflecting the vast diversity of genetic risk^20^. Acting alone or in combination, such changes may lead to local and long-range degradation of the ordered local and global connectivity and functional synchrony of neuronal networks needed to support the cognitive and perceptual operations, and hippocampal-dependent forms of memory (such as episodic or spatial working memory) affected in SCZ^21,22^. Despite the onset of SCZ-associated psychosis in adolescence or adulthood, schizophrenia also has a strong neurodevelopmental component much before the presentation of psychotic symptoms. Therefore, it is critical to investigate the neuronal deficiencies in development as well as their manifestation in the mature functional networks.

We aimed to develop in vitro models of SCZ-related network pathophysiology by utilizing two well-characterized genetically engineered mouse models of SCZ-risk genes^11,14,23–25^. First, *Setd1a*+*/−* mice, which model loss-of-function mutation in *SETD1A*, a lysine-methyltransferase that modulates the expression of a large number of genes expressed brain-wide but especially in the neocortex^24^, in large part by mediating methylation on lysine 4 on the histone H3 protein (H3K4)^26,27^. Second, *Df(16)A*+*/*− mice, which model a highly penetrant microdeletion in human chromosome *22q11.2* locus resulting in sporadic cases of SCZ in ~30% of carriers^28^. Both mutations show the strongest association with SCZ in respective exome sequencing and structural variant scans agnostic to the genotype^29–31^ (https://schema.broadinstitute.org/results) and, while mutation carriers exhibit additional behavioral and cognitive symptoms, SCZ is the most prominent psychiatric diagnosis for both mutations. These genetic models were recently shown to recapitulate the cognitive and circuitry deficits generally associated with SCZ^11,14,23,24,32,33^. We derived MoNNet (SCZ-MoNNet) preparations from these systems, and performed exhaustive comparative characterization over several weeks, revealing significant alterations in the stability of modules/ensembles formation, much-reduced global network synchrony with much smaller impact on local synchrony, and lack of hierarchical modular organization due to severely reduced inter-modular functional interactions. To reveal the underlying molecular mechanisms, we performed RNAseq experiments for MoNNets derived from *Setd1a*+*/−* and WT siblings. The comparative analysis of gene expressions in *Setd1a*+/− vs. WT sibling MoNNets revealed dysregulation of cytoskeleton/neuronal structure and synapse signaling/function-related genes, in striking correlation with the alterations in structural connectivity, synaptic plasticity and neuromodulation generally associated with SCZ^18,19^. Furthermore, we demonstrate the utility of this approach for drug discovery by performing retrospectively predictive pharmacological rescue experiments with antagonists of LSD1 demethylase activity (ORY-1001 and TCP) which were recently shown to effectively rescue some of the cognitive behavioral abnormalities in *Setd1a*+*/−* mice^24^. We found that treatment with these compounds promoted stabilization of functional connections and modules in the later mature stages, revealing their network-level mechanisms of action.

In summary, we report the development and extensive characterization of highly complex modular neuronal networks (MoNNet), and their utility in developing quantitative in vitro models of some of the functional network dysfunctions known to be associated with complex brain disorders like SCZ^18,19^.

## Results

### MoNNets recapitulate aspects of the brain functional network properties

**Fig. 1:**
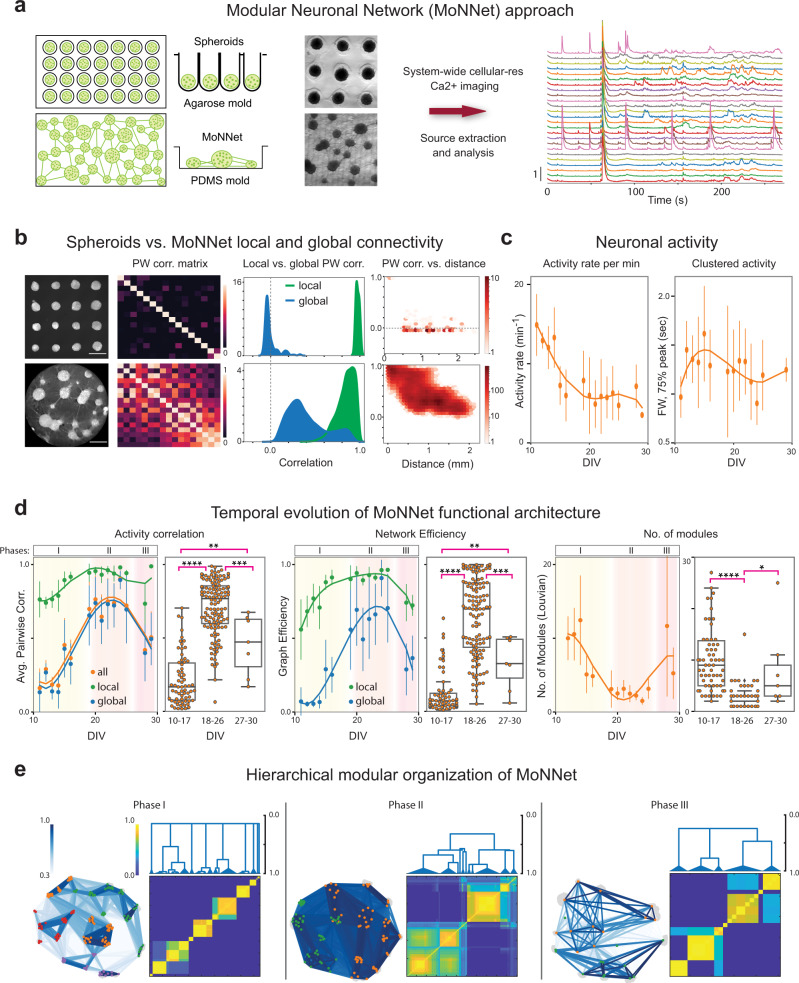
Characterization of Modular Neuronal Network (MoNNet) approach. **a** Schematic overview of MoNNet approach. Extracted embryonic hippocampal cells were infected with AAV1.Syn.GCaMP6f.WPRE.SV40, and plated on a PDMS mold for self-organized assembly of MoNNet, or on an agarose mold to generate spheroids. System-wide cellular-resolution Ca^2+^ imaging was performed at 30 Hz. **b** Comparison of local (i.e. neuron pairs within same spheroids) and global (i.e. across spheroids) functional connectivity in spheroids vs. MoNNet. Left to right: representative images; pairwise (PW) correlation matrix of aggregated activities of spheroids; neuronal PW correlation histograms; density plot for correlation vs. distance. Also see Supplementary Fig. 1 and Video 1 for representative raster plots and neuronal activity, and Supplementary Fig. 2 for PW correlation vs. distance relationships, and for distribution of spheroids sizes and distances among nearest neighbors in MoNNets. These representative comparisons were independently repeated >10 times. **c**, **d** Temporal evolution of MoNNet functional properties assessed by Ca^2+^ imaging of 282 different MoNNet samples. Note that each sample was only imaged once to minimize image acquisition associated artifacts and non-active samples were filtered. In all graphs, biologically independent samples (n) across DIVs as follows: *n*(11): 7, *n*(12): 5, *n*(13): 9, *n*(14): 27, *n*(15): 11, *n*(16): 7, *n*(19): 28, *n*(20): 33, *n*(21): 14, *n*(22): 29, *n*(23): 9, *n*(24): 10, *n*(25): 7, *n*(28): 3, *n*(29): 4. **c** Activity rates per minute and Full width at 75% of peak. **d** Left-to-right: average PW correlation of local (green), global (blue) and all (orange) neuron pairs; distribution of average global PW correlation in three phases; local and global network efficiency of weighted functional graphs; number of detected modules. Statistical significance was calculated by one-way ANOVA with post-hoc Tukey’s test for multiple comparisons (*padj < 0.05, **padj < 0.01, ***padj < 0.001, ****padj < 0.0001). padj (Activity Correlation) for DIV 10–17 & 18–26: 1.8e−43, for 10–17 & 27–30: 0.00098, for 18–26 & 27–30: 0.00052. padj (Network Efficiency) for DIV 10–17 & 18–26: 3.4e−34, for 10–17 & 27–30: 0.0004, for 18–26 & 27–30: 0.0026. padj (No. of Modules) for DIV 10–17 & 18–26: 3.2e−21, for 18–26 & 27–30: 1.6e−8. Also see Supplementary Fig. 2 for comparisons of all the measures across the three phases, Supplementary Videos 2, 3 for example recordings of MoNNets and spheroids from the three phases, and Supplementary Video 4 for older (DIV 49 and 64) MoNNet samples. The error bars are std. deviations and center points are mean values. The boxplots show minimum, first quartile, median and third quartile. **e** Representative examples of hierarchical modular organization of MoNNets. Left-to-right: MoNNet functional graph with nodes in same modules colored same, and co-classification (into the same module) probability heat maps and their consensus clustering dendrograms. Also see Supplementary Fig. 3 for results from multiple edge weight thresholds. For all plots, error bars are std. deviations and center points are mean. The error bars in histograms are 95% confidence interval. All scale bars are 500 μm. Source data are provided as a Source Data file.

We developed and characterized the modular neuronal network (MoNNet) approach by utilizing mouse embryonic (E17–18) hippocampal cells, taking advantage of their faster developmental timescale and extensive knowledge of in vivo network properties. The dissociated cells were infected with the AAV1.Syn.GCaMP6f.WPRE.SV40 vector for synapsin promoter-driven GCaMP6f expression in neurons and were plated on a non-adhesive polydimethylsiloxane (PDMS) mold for generating MoNNets, and on an agarose mold to generate individual isolated spheroids for comparative characterization (Fig. 1a; preparation and culture health validation details in “Methods”). The self-organization of MoNNets started with spontaneous formation of spheroids units, followed by their expansion and development of interconnections to result in physically modular 3D networks of cohesive spheroids of cells (Supplementary Fig. 1, 2). Lack of physical constraints in PDMS molds resulted in spheroids of varied sizes and interconnections as quantified in Supplementary Fig 2b. Next, we performed cellular-resolution live imaging (~4.5 min, 30 Hz) of these preparations for capturing system-wide Ca^2+^ signaling as a proxy for neuronal activities (imaging details described in “Methods”).

We first compared the neuronal activity synchronization in individual spheroid preparations and the MoNNet assemblies, to assess the local (i.e., neuron pairs belonging to same physical spheroid unit) and global (i.e., neuron pairs belonging to different physical spheroid unit) functional connectivity over time. As shown in Fig. 1b, Supplementary Fig. 1 and Videos 1–3, the neurons in individual unconnected spheroids exhibited high local activity correlation and no global connectivity, suggesting an absence of cell-culture media driven signaling. The neuronal activity in MoNNet became highly correlated in local pairs, but exhibited varied strength in global correlations following an inverse scaling relationship with the physical distances, reminiscent of in vivo observations^16^. These results suggest that the neurons in MoNNets can indeed form local and global functional connections throughout the network.

To systematically characterize the dynamics of MoNNet activity, we acquired system-wide cellular resolution Ca^2+^ imaging data from 282 different MoNNet samples, densely covering time-points up to 30 days in vitro (DIV). The MoNNet preparations remain stable and healthy for several months (representative example recordings of DIVs 49 and 64 MoNNets in Supplementary Video 4). As shown in Fig. 1c, the neuronal activity rates of MoNNets were higher in the beginning with a gradual decline over time, similar to developing brain networks. And, the neuronal activity duration, as quantified by the full width at 75% of the peak of ∂F/F traces, showed tendency to increase in the early DIVs, and then decrease over time (in resemblance to the activity motifs described in the hippocampus recordings^34^). Next, we calculated the average pairwise activity correlations of all the MoNNet samples to map the network activity synchronization. As shown in Fig. 1d (also Supplementary Fig. 2 and Video 2), the activity correlation of local neuronal pairs remained very high throughout, whereas the global functional connectivity followed an approximate 3-phase dynamics: low activity correlation initially (i.e., pre-synchronization phase), transition to high synchronization (i.e. synchronized phase), and eventual diversification into hierarchically organized modules (i.e., post-synchronization phase). This temporal profile, at a macro level, is consistent with the process of neural circuit maturation by synchronization and diversification into modules. However, the exact similarities at the level of cellular mechanisms need to be investigated in further studies. We approximated the phase boundaries at half of maximum average pairwise correlation profile peak (Fig. 1d). In contrast, the neuronal pairwise co-activity within single unconnected spheroids remained very high throughout (Supplementary Video 3).

We utilized graph theory methods to further investigate the evolving functional networks in MoNNets. We derived functional connectivity graphs by representing neurons as nodes and their activity correlation as weighted edges. First, we calculated graph efficiency, which is a widely used measure of overall network connectivity. Similar to the activity correlation, the local graph efficiency rapidly increased and remained high throughout, whereas the global efficiency followed the same 3-phase temporal progression (Fig. 1d, Supplementary Fig. 2d). Next, we analyzed the modular architecture of the MoNNets by applying Louvain community detection method^35^ to estimate the number of distinct modules. As shown in Fig. 1d and Supplementary Fig. 2d, MoNNet in phase I consists of a large number of modules, which merge in phase II, and eventually diversify into hierarchical clusters of modules in Phase III. The neurons belonging to the same spheroids largely belong to the same modules, as would be expected from high local activity correlation. These phases are significantly different from each other, as shown by several neuronal and network properties in Fig. 1d and Supplementary Fig. 2. Figure 1e and Supplementary Fig. 3 show representative examples from three phases, visualizing the hierarchical modular graphs and co-classification matrix^36^, which measures the probability of pairs of neurons co-classifying as part of the same module, and the consensus clustering dendrograms (detailed in the “Methods” section).

Overall, these results demonstrate that MoNNet recapitulates aspects of the brain functional network properties such as segregated local-global synchrony, formation and maintenance of ensembles (discussed later in further details) and hierarchical modular organization, thus may provide a quantitative in vitro platform for modeling of network dysfunctions associated with complex brain disorders like SCZ.

### Pharmacological characterization of neuronal activity and network synchrony in MoNNet

**Fig. 2:**
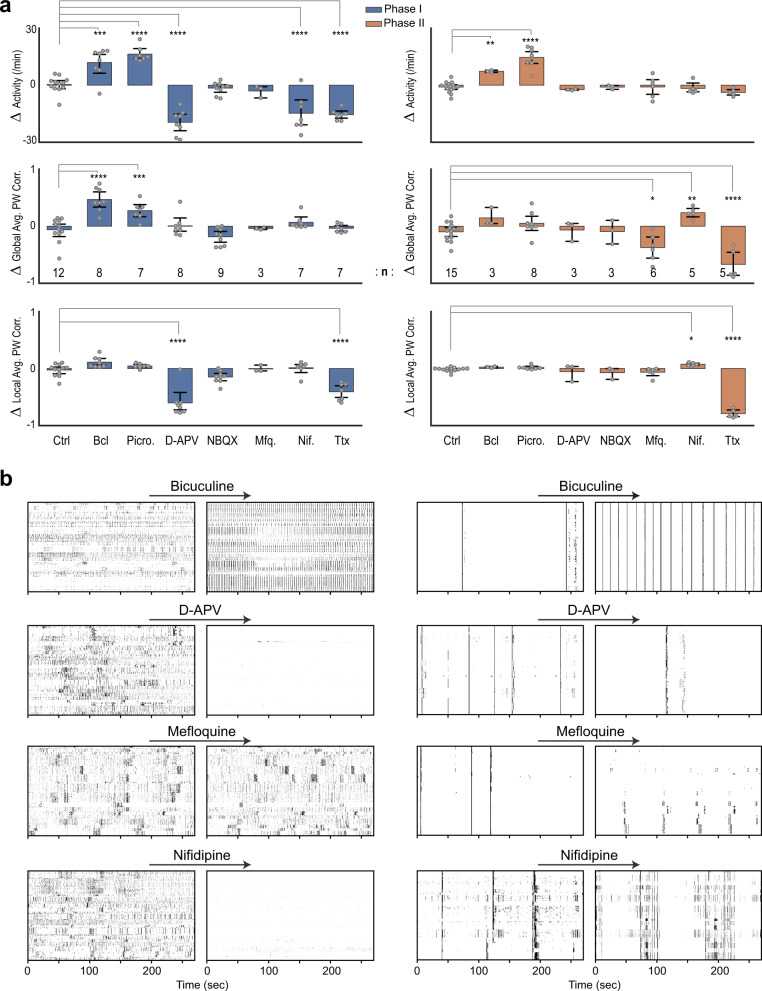
Pharmacological characterization of MoNNet neuronal and network activity. **a** Characterization of MoNNet functional properties by pharmacological treatments with GABAergic and Glutamatergic synaptic inhibitors: Bicuculine [10 μM], Picrotoxin[10 μM], D-APV [40 μM] and NBQX [10 μM]), and ionic channel blockers: Mefloquine [25 μM], Nifedipine [10 μM] and TTX [1 μM]. Control samples were treated with the solvent DMSO alone. *Y*-axes quantify the increment relative to the baseline in same samples, after 1 h of treatments. Number of biological replicates (*n*) is shown on the plot. Blue and Orange bars are for MoNNets in phase I and II, respectively. Statistical significance was calculated by using one-way ANOVA and Dunnett multiple comparison test. * padj < 0.05, **padj < 0.01, ***padj < 0.001, ****padj < 0.0001. Bar heights are mean, and the error bars are 95% confidence interval. All sample replicates are biological. **b** Representative raster plots before (baseline) and after treatments. Left and right columns are for phase I and II, respectively. Also see Supplementary Figs. 4, 5 for complete set of representative raster plots, and Supplementary Videos 5, 6 for representative recordings before and after pharmacological treatments. Source data are provided as a Source Data file.

We performed pharmacological treatments of MoNNets with widely used antagonists of synaptic activity (bicuculine, picrotoxin, D-APV, and NBQX) and ion channels (TTX, nifedipine, and mefloquine). All experiments were performed with MoNNets in phases I, and also in phase II to investigate the underlying molecular and physiological mechanisms involved in MoNNet maturation. The neuronal activity was captured by system-wide cellular resolution imaging before (baseline) and after 1-hour pharmacological treatments. We used a custom data analysis pipeline for extracting the neuronal activity sources, and to calculate changes in the following parameters: neuronal activity rates, global and local average pairwise co-activity (Fig. 2 and Supplementary Figs. 4–6 and Videos 5, 6). To mitigate the sample-to-sample variabilities, effect of pharmacological treatments was quantified relative to the baseline values in the exact same samples (Fig. 2a, Δ = after—before treatment).

Synaptic transmission blockers were used to investigate the role of excitatory and inhibitory neurons in MoNNet functional properties. As shown in Fig. 2, both bicuculine and picrotoxin (GABA-A receptor antagonists) treatments of Phase I MoNNet increased the neuronal activity and local and global average pairwise correlations. Similarly, MoNNets in Phase II, which have higher baseline activity correlations, showed increased neuronal activity and slightly increased global average pairwise correlation (see Supplementary Fig. 6 for assays with multiple concentrations of bicuculine and picrotoxin). These results show the existence of inhibitory neuronal connections and their role in shaping MoNNet maturation. Next, treatments with glutamatergic transmission inhibitor D-APV (NMDA Receptor antagonist) resulted in complete silencing of the MoNNet activity in phase I, and slight reduction of activity in phase II. However, another inhibitor of glutamatergic transmission NBQX (AMPA and Kainate Receptor antagonist) did not alter the neuronal activity rate, but showed a small decrease in both global and local network activity correlation. These results suggest the role of NMDA receptors but not AMPA and Kainate receptors in driving neuronal activity, and potential role for AMPA and Kainate receptors in the refining functional connection stability.

The role of ion channels in MoNNet functionality was assessed by treatments with Mefloquine, which is an antagonist of Cx36 and Cx50 gap channels; Nifedipine, an L-Type Calcium channel blocker; and Tetrodotoxin (TTX), which is a blocker of sodium channels. As shown in Fig. 2, Mefloquine treatment resulted in a significant reduction in global network synchrony in phase II, but not in phase I, suggesting the emergence of gap-junction driven connections over time. Next, we used Nifedipine to assess the role of L-Type calcium channels, and found decreased neuronal activity in phase I, but not in phase II. Whereas, the global as well as local activity correlation was increased in phase II. The blocking of sodium channels with TTX resulted in reduced activity, however, some residual activity remained in phase I, suggesting the existence of spontaneous non-synaptic calcium activity. These results suggest increased role of ion channels over time in the development and maintenance of neuronal activity and synchrony.

Altogether, these systematic pharmacological experiments demonstrate the existence of complex excitatory, inhibitory and gap-junction driven activities and neuronal plasticity in MoNNets, similar to in vivo complex neural circuits. The synaptic transmission of glutamatergic neurons, via NMDA receptors but not AMPA and Kainate receptors, may play a major role in driving the network activity, whereas AMPA and Kainate receptors also played a role in the establishment of functional connections. The existence of inhibitory GABAergic transmission within and across spheroid units facilitate the establishment of complex activity patterns (consistent with the electrophysiology recordings discussed in the next sections). Finally, the emergence of functional gap junctions over time promotes global synchronization.

### Cellular architecture of MoNNets

**Fig. 3:**
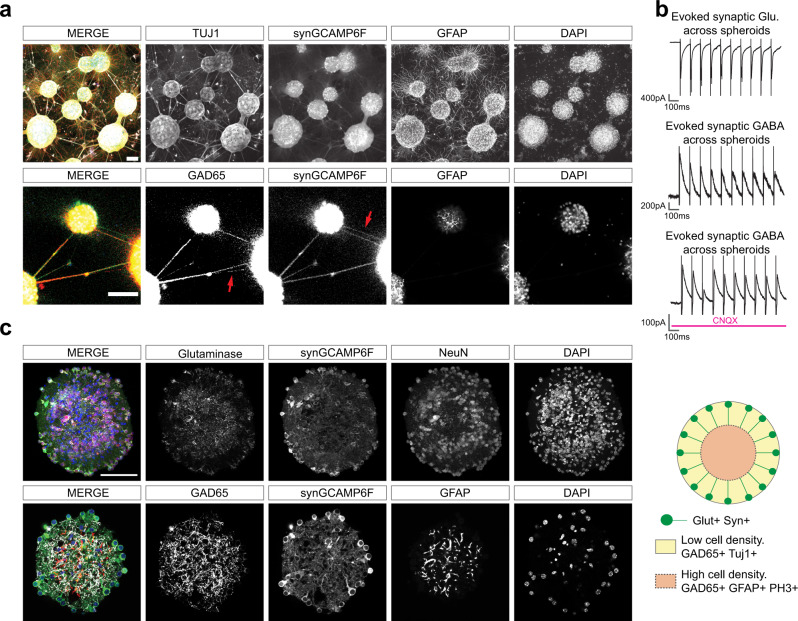
Characterizing the cellular architecture of MoNNet. **a** Whole-mount immunostaining of four weeks old MoNNets. Top row: maxima projections showing Tuj1, Syn-GCaMP6f, GFAP and DAPI labeling. Bottom row: a confocal optical section showing co- or exclusive (red arrow) labeling of interconnections with GAD65 or synGCaMP6f signals, suggesting existence of excitatory as well as inhibitory signaling transmission across spheroids unit in MoNNets. Scale bars are 100 μm. These representative experiments were independently repeated >3 times. **b** Electrophysiology recordings validating the existence of monosynaptic functional glutamatergic and GABAergic signal transmission across spheroids. For all recordings, a neighboring spheroid, located ~250–300 μm, away was stimulated at 10 Hz. Top-to-bottom: representative 1 s trace of a cell held at −70 mV showing robust evoked excitatory synaptic transmission across spheroids; representative 1 s trace of a cell held at 0 mV showing robust evoked inhibitory synaptic transmission across spheroids; representative 1 s trace of a cell held at 0 mV showing robust evoked inhibitory synaptic transmission during CNQX (10 μM) blockade of glutamatergic transmission. **c**, Immunostaining of 2 weeks old individual spheroids vibratome sections (50 μm thick) to visualize their cellular architecture. Glutaminase and NeuN immunolabeling revealed preferentially peripheral localization of glutamatergic cells, whereas the GAD65 and GFAP expression is preferentially localized in the inner regions. A schematic summary is shown on right. Scale bars are 100 μm. These representative experiments were independently repeated >3 times. Also see Supplementary Fig. 7 for additional GAD65 stainings, Supplementary Fig. 8 for PH3 and activated Caspase3 labeling, and Supplementary Video 7 for Confocal z-stack showing the 3D cellular architecture of MoNNet.

We performed immunolabeling, RNAseq (discussed in the next section) and electrophysiology experiments to assess the existence and distribution of major cell types in MoNNet. We first analyzed the MoNNet connectivity by performing whole-mount immunolabelling of MoNNets with antibodies against Tuj1 (neuronal marker), GAD65 (inhibitory neurons marker) and GFAP (glial marker). As shown in Supplementary Video 7, the MoNNets are inter-connected with Tuj1+ axonal fiber bundles that are truly organized in 3D space. Some of these inter-spheroid connections are also positive for GAD65, suggesting the existence of excitatory and also some inhibitory axonal fibers (Fig. 3a, Supplementary Fig. 7), consistent with pharmacological inhibition results described in the previous section. Additionally, we found that while GFAP+ cells are localized within spheroids, on the bottom surface of the PDMS mold and also in the thicker inter-connecting axonal bundles (Supplementary Video 7).

Next, we performed electrophysiology experiments to test the functionality of Glutamatergic and GABAergic interconnections in MoNNets. As shown in Supplementary Fig. 8, we found the existence of spontaneous glutamatergic and GABAergic action potentials in MoNNets. Further, we recorded evoked responses from neurons in a spheroid, while simultaneously stimulating (10 Hz) a neighboring spheroid located at least 250–300 μm away. As shown in Fig. 3b, we found evoked Glutamatergic and GABAergic responses, the latter of which persisted even in the presence of AMPA/Kainate antagonist CNQX (similar to the pharmacological inhibitions results with NBQX treatments, Fig. 2), strongly suggesting the existence of monosynaptic excitatory and inhibitory connections across the spheroid units, consistent with GAD65 immunostainings (Supplementary Fig. 7).

We also performed staining and imaging of middle vibratome sections (50 µm) derived from individual spheroids (2 weeks old; phase I). This approach allowed us to bypass the limitations of reagents penetration of whole-mount preparations. As shown in Fig. 3c, immunostaining of Glutaminase (excitatory neuron marker), NeuN (neuronal marker) and Synapsin promoter-driven GCamP6f expression, showed that the excitatory cells are largely located in the periphery, whereas, the expression of GAD65 and GFAP is preferentially localized to the inner zones. Additionally, as shown in Supplementary Fig. 8, the general lack of activated Caspase-3 immunolabelling suggested an absence of significant apoptosis, and the PH3 + immunolabelling revealed preferential localization of mitotic cells to the inner zone.

Altogether, these results suggest the existence of excitatory as well as inhibitory structural and functional connections across individual spheroids in MoNNets, and a specific layered arrangement of cell types (as opposed to a disorganized mixture of cells), consistent with the functional complexity of MoNNets.

### Comparative gene expression profiling of MoNNets and spheroids

**Fig. 4:**
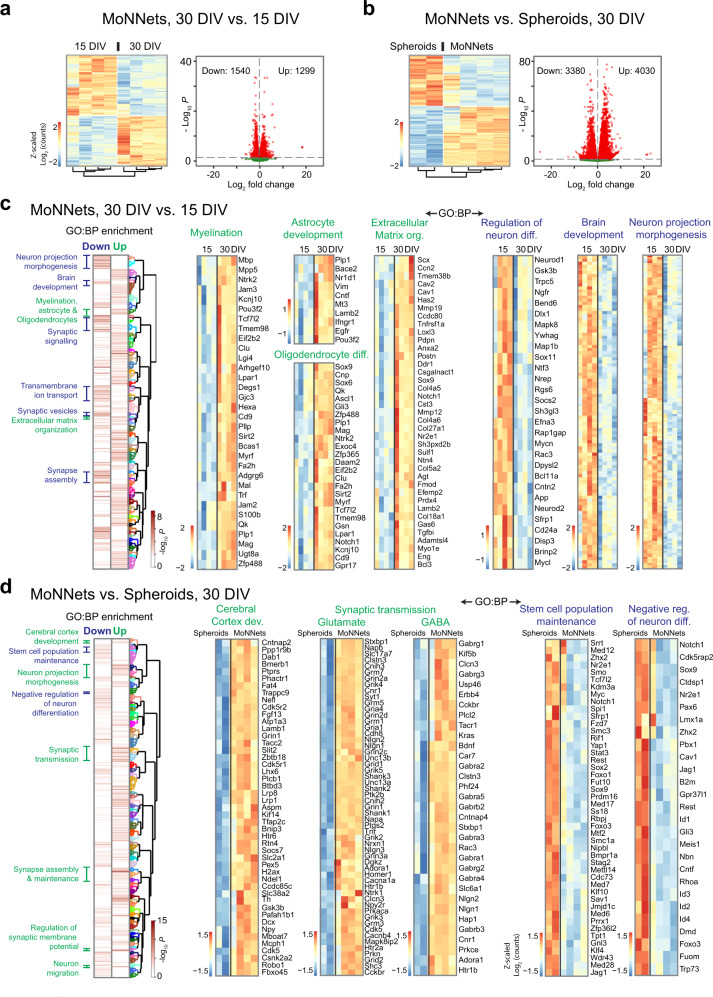
Characterizing the gene expression differences in MoNNets and spheroids. **a** Heat map and volcano plot of differential gene expression (DESeq2, padj < 0.05) in MoNNets at DIV30 relative to DIV15. **b** Heat map and volcano plot of differential gene expression (padj < 0.05) in MoNNets relative to spheroids at DIV30. **c** Comparative enrichment of Gene Ontology: Biological Process (GO:BP) terms in up (green) and down (blue) regulated genes in MoNNets at DIV30, relative to DIV15. Gene expression heat maps are shown for specific GO:BP terms. Also see Supplementary Fig. 9 for gene names associated with “Brain development” and “Neuron projection morphogenesis” terms. **d** Comparative enrichment of GO:BP terms in up (green) and down (blue) regulated genes in MoNNets, relative to spheroids. All sample replicates are biological. Also see Supplementary Fig. 10 for details of GO:BP terms clusters. Gene expression heat maps are shown for specific GO:BP terms.

We performed total RNAseq experiments on MoNNet samples at DIV15 and DIV30, and spheroid samples at DIV30 (Fig. 4a, b). As shown in Fig. 4a, we found 1299 up and 1540 downregulated genes (padj < 0.05) in DIV30 MoNNets, relative to early DIV15 MoNNets stage, and 4030 over and 3380 under-expressed genes (padj < 0.05) relative to spheroids at DIV30. First, we performed Gene Ontology enrichment comparison (by using topGO^37^ and ViSEAGO^38^ packages in R) in up and downregulated genes in DIV30 MoNNets, relative to DIV15 MoNNets. We found significant enrichment (*p* < 0.01) of GO:BP terms related to Myelination (*p* = 0.0002, GO:0042552), Astrocyte development (*p* = 0.0048, GO:0014002), Oligodendrocyte differentiation (*p* = 0.0003, GO:0048709) and Extracellular matrix (ECM) (*p* = 6.7×10^−5^, GO:0030198) in upregulated genes in DIV30 MoNNets (Fig. 4c, Supplementary Fig. 9), providing support for expression of some of the genes relevant for myelin sheaths, astrocytes (also supported by immunostaining with GFAP, shown in Fig. 3c and Supplementary Video 7) and extracellular matrix components (ECM) in maturing MoNNets. For example, we found significant upregulation of key myelin development and differentiation markers (Fig. 4c) including *Myrf* (Myelin regulatory factor), *Mbp* (Myelin basic protein), *Mag* (Myelin associated glycoprotein), *Mal* (Myelin and lymphocyte protein), *Mpp5*, and *Bcas1*; genes important for Oligodendrocytes development and differentiation, including *Ascl1*, *Sox6*, *Sox9*, *Zfp488*, *Qk*, *Sirt2* and *Gsn*; and highly specific marker genes for astrocytes including *Vim*, *Cntf*, *Egfr*, *Bace2*, *Plp1,* and *Lamb2* (Laminin, beta 2). Strikingly, we also observed high expression of extracellular matrix-related genes such as *Mmp12*, *Mmp19*, *Col27a1*, *Col5a2,* and *Sox9*. These results suggest the over-expression of genes involved in myelination, astrocytes and ECM in DIV30 MoNNets.

In contrast, the downregulated genes in DIV30 MoNNets (i.e. upregulated in DIV15 MoNNets) were enriched in GO:BP terms brain development (*p* = 0.005, GO:0007420), regulation of neuron differentiation (*p* = 0.008, GO:0045664) and neuron projection morphogenesis (*p* = 0.003, GO:0048812), reflecting the matured developmental stage at DIV30. For example, we observed significant downregulation of genes important for early brain development and neuronal differentiation (Fig. 4c, Supplementary Fig. 9) including morphogen signaling genes such as *Wnt2*, *Wnt3*, *Fgf10*, *Fgf13*, *Gsk3b,* and *Sfrp1*; key development and differentiation transcription factors such as *Lhx2*, *Dlx1*, *Lhx9*, *Klf7*, *Neurod1*, *NeuroD2*, *NeuroD6*, *Tbr1*, *Nanos1*, *Sox11*, *Bcl11*, and *Cux2*; growth factors, neuronal migration, and axon guidance genes such as *Ngfr*, *Ntf3*, *Dcx*, *Robo1*, *Slit2*, *Slit3*, *Dscam*, *Nlgn3*, *Nrxn1* and *Rtn4rl2*; and GTPase *Rac3* (see Supplementary Fig. 9 for complete list). In addition, consistent with decreased synchronization and neuronal activity rate over time in maturing brains and MoNNets (Fig. 1c), we found downregulation of genes involved with synapse assembly and signaling in MoNNets at DIV30, relative to DIV15. Overall, the MoNNets gene expression differences in early and late stages suggest over-expression of genes involved in network maturation processes such as myelination.

Next, we compared the gene expression profile of MoNNets with single spheroid preparations of exact same age, DIV30. As shown in Fig. 4d and Supplementary Fig. 10, the over-expressed genes in MoNNets, relative to spheroids, were highly enriched in GO:BP terms synaptic signal transmission (including glutamatergic (*p* = 6.06 × 10^−10^, GO:0035249) and GABAergic (*p* = 3.19 × 10^−7^, GO:0051932), cerebral cortex (*p* = 0.001, GO:0021987), and terms associated with synapse organization, dendrites, and axons morphogenesis (shown in detailed heat maps in Supplementary Fig. 10). For example, several genes required for glutamatergic and GABAergic synaptic signaling pathways were highly over-expressed in MoNNets compared to spheroids (gene expression heat maps in Fig. 4d). In contrast, the under-expressed genes in MoNNets (i.e. over-expressed in spheroids), were enriched in GO:BP terms associated with stem cell property maintenance (*p* = 4.9 × 10^−5^, GO:0019827; e.g. *Foxo1*, *Foxo3*, *Klf4*, *Klf10*, *Sox2*, *Sox9*), negative regulators of neuron differentiation (*p* = 0.0005, GO:0045665; e.g. *Meis1*, *Lmx1a*, *Zhx2*, *Nr2e1*, *Gli3*, *Id1*, *Id2*, *Id3*, *Id4*, *Pax6*) and early embryonic development processes (shown in heat maps in Fig. 4d), suggesting much more differentiated and matured state for MoNNets compared to single spheroids. Overall, these differences in gene expressions in MoNNets and spheroids reflect the improvements in connectivity-dependent MoNNets cellular complexity, consistent with the observed differences in their functional network complexities (Fig. 1). The formation of short- and long-range neuronal connections and, as a result, enhanced and diverse neuronal activity patterns in MoNNets may be significantly contributing to their maturation^39^.

### Modeling SCZ-related network abnormalities with *Setd1a*+*/−* and *Df(16)A*+*/−* MoNNets

Building upon the strengths of MoNNet systems for in vitro modeling of complex network activity patterns and properties of brain networks, we aimed to establish high-fidelity in vitro models of SCZ-related network function abnormalities. Several recent studies in humans and model organisms have revealed a number of molecular, cellular and functional alterations emerging as a result of genetic liability, which in turn lead to network dysconnectivity and synchrony instability^11,14,23,33^, including deficits in ordered activity and plasticity of hippocampal neuronal assemblies. However, a comprehensive system-wide circuit-level understanding remains limited, in large part due to the technical limitations of cellular-resolution mapping of system-wide activity.

We aimed to develop in vitro models of network abnormalities caused by two highly penetrant SCZ-risk mutations: *Setd1a*+*/−* which models highly penetrant loss-of-function mutations in *SETD1A*, a confirmed SCZ-risk gene encoding a lysine-methyltransferase and *Df(16)A*+*/*− which models a highly penetrant deletion at the *22q11.2* locus, one of the largest known genetic risk factors for SCZ. The animal models of these mutations have been shown to generally recapitulate the SCZ-associated deficiencies in behaviors and neural circuits. We prepared MoNNets from these genetic models (collectively termed SCZ-MoNNet for ease of discussion) and their WT siblings to exhaustively compare and characterize various quantitative measures of network functional synchrony. A total of 488 unique MoNNet samples were generated and analyzed. Similar to other experiments, we performed system-level cellular-resolution Ca^2+^ imaging and comparative analysis. As these disease mice models were generated in C57BL/6J background, we compared the overall temporal progression of synchrony in C57BL/6J (WT siblings) vs. CD-1 WT strains (Supplementary Fig. 11), showing similar profiles with a slight temporal shift to right in C57BL/6J. Also, we found the overall morphology of SCZ-MoNNets to be very similar to WT sibling MoNNets (Video 8).

**Fig. 5:**
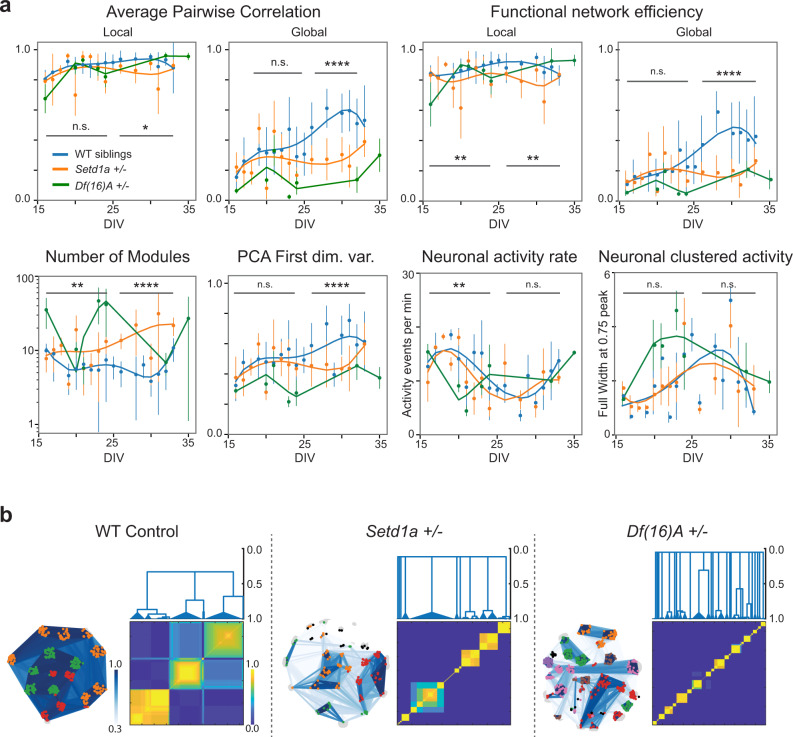
Characterization of in vitro models of SCZ-associated network pathophysiology. **a** Comparison of various local and global quantitative measures of MoNNets derived from *Setd1a*+/− (orange), WT siblings(blue) and *Df(16)A*+/− (green): average pairwise correlation, global efficiency of functional weighted graphs, number of detected modules, activity data variance captured by the first PCA dimension, predicted activity-onset events rate per minute; full width at 75% of the maximum peak of ∂F/F traces. Statistical significance was calculated by comparing pooled data (black bars) of *Setd1a*+/− vs. WT siblings. Statistical significance (two-sided t-test) is defined as: *p < 0.05, **p < 0.01, ***p < 0.001, ****p < 0.0001. For WT control siblings, 272 different MoNNet samples were imaged. Note that each sample was only imaged once to minimize image acquisition associated artefacts and non-active samples were filtered. Biologically independent samples (n) across DIVs in graph as follows. *n*(16): 12, *n*(17): 6, *n*(19): 7, *n*(20): 8, *n*(21): 14, *n*(22): 13, *n*(23): 12, *n*(24): 14, *n*(25): 6, *n*(26): 7, *n*(28): 7, *n*(29): 8, *n*(30): 6, *n*(31): 9, *n*(32): 9, *n*(33): 5. For *Setd1a*+*/−* 158 different MoNNet samples were imaged. Each sample was only imaged once, and non-active samples were filtered. Biologically independent samples (n) across DIVs in graph as follows. *n*(16): 12, *n*(17): 5, *n*(18): 3, *n*(19): 6, *n*(20): 8, *n*(21): 6, *n*(22): 14, *n*(23): 12, *n*(24): 5, *n*(26): 5, *n*(28): 5, *n*(30): 7, *n*(31): 7, *n*(33): 3. For *Df(16)A*+/−, 58 different samples were imaged. Each individual sample was imaged only once and non-active samples were filtered. Biologically independent samples (n) across DIVs in graph as follows. *n*(16): 4, *n*(20): 3, *n*(21): 9, *n*(23): 4, *n*(24): 8, *n*(32): 8, *n*(35): 4. **b** Representative examples of comparing hierarchical modular organization. Nodes belonging to same module are colored same. Co-classification matrix heatmap and clustering dendograms are shown to visualize hierarchical relationship. For all plots, mean and std. deviation are plotted. Also see Supplementary Fig. 11 and Video 8. Source data are provided as a Source Data file.

As shown in Fig. 5a, the global functional synchrony was strongly reduced in SCZ-MoNNets in later phases, and a much smaller decrease was observed in local synchrony. Similarly, the global network efficiency was also highly diminished in SCZ-MoNNets in the latter stages while the local network efficiency showed smaller reductions. These results reveal a much more prominent role of the global synchrony alterations in causing SCZ-like network dysfunction, further extending the mechanistic insights from in vivo 2-photon calcium imaging studies of parts of cortical circuits^11,14,23,33^. Next, we calculated the number of modules in SCZ-MoNNets and their WT siblings using the Louvain community detection method, as described in previous sections. As shown in Fig. 5a (and Supplementary Video 8), we found a large increase in the number of modules in SCZ-MoNNets, compared to the WT siblings, throughout the observation period. This is in strong agreement with the observed reduction of global synchrony resulting in isolated unstable modules. The neuronal activity rates, and clustered activity generally remained similar with a slight reduction observed in early phases (Fig. 5a). Figure 5b shows representative examples comparing the organization of modules in SCZ-MoNNet and WT siblings, demonstrating the existence of much smaller modules with striking loss in inter-modular communication. Although both *Df(16)A*+*/−* and *Setd1a*+*/−* SCZ-MoNNets showed remarkably similar temporal patterns of network dysfunctions, the defects are much more severe in *Df(16)A*+*/−* MoNNets, recapitulating in vivo observations that mouse models of highly penetrant multi-genic CNVs (such as the *Df(16)A*+*/−* mice) exhibit markedly more severe behavioral phenotypes than mutant mice carrying single-gene mutations (such as the *Setd1a*+*/−* mice)^12^. Overall, the system-wide network analyses provide strong support for defects in global synchrony and modular stability as a significant contributing factors for SCZ-associated network dysfunction, in resemblance with in vivo observations.

### Comparative gene expression profiling of *Setd1a*+*/−* and WT sibling control MoNNets

**Fig. 6:**
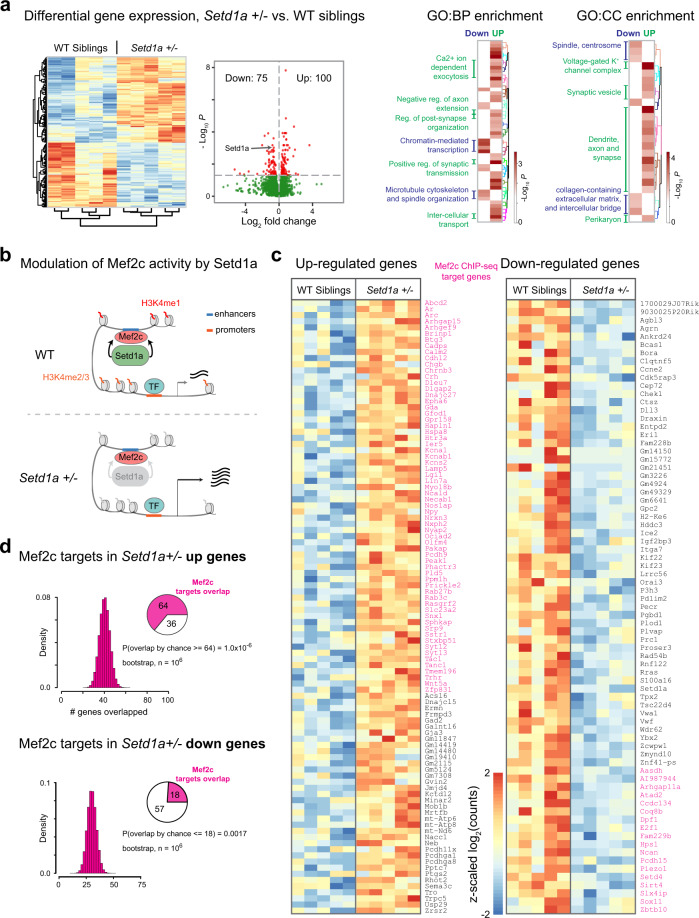
Characterization of gene expression differences in *Setd1a*+*/−* and WT siblings. **a** Heat map and volcano plot of differential gene expression (DESeq2, padj < 0.05) in *Setd1a*+*/−* relative to the WT siblings (DIV 30). Comparative enrichment of Gene Ontology: Biological Process (GO:BP) and Cellular Components (GO:CC) terms is shown for up (green) and down (blue) regulated genes. Also see Supplementary Fig. 12 for complete list. **b** Schematics summarizing the modulation of Mef2c activity by Setd1a. **c** Gene expression heat maps are shown for up- and down- regulated genes. Magenta-colored genes are also known targets from Mef2c ChIP-seq studies. **d** Bootstrap sampling (10^6^) for estimating statistical significance of overlap with Mef2c targets by chance. P-values are uncorrected probabilities as defined in the panel. All sample replicates are biological.

We performed RNAseq experiments to compare the gene expression levels in MoNNets derived from *Setd1a*+*/−* and WT siblings. As shown in Fig. 6a, we found statistically significant (padj < 0.05) upregulation of 100 and downregulation of 75 genes in *Setd1a*−/+ MoNNets, relative WT sibling controls. The differential gene expression analysis recapitulated the expected decrease in *Setd1a* expression (Fig. 6a). First, we performed comparative Gene Ontology enrichment analysis of up and downregulated gene sets. As shown in Fig. 6a and Supplementary Fig. 12, the upregulated genes are enriched in GO:BP terms associated with synapse signaling and organization, and Ca^2+^ dependent exocytosis, and the GO:CC terms associated with synaptic vesicles, dendrites, axons, and voltage-gated K^+^ ion channel complex (details of enriched GO:BP and GO:CC terms in Supplementary Fig. 12). The downregulated gene set is enriched in microtubule-dependent cytoskeleton, spindle organization and chromatin-mediated transcription related GO:BP terms, and collagen-containing extracellular matrix, intercellular bridge, and spindle related GO:CC terms.

The upregulation of the synapse-related genes in *Setd1a*+*/−* may suggest alterations in activity-dependent modulation of synaptic plasticity (similar to in vivo observations^18,19^), thereby affecting the functional connection stability. For example: *Kcns2*, *Kcnb1,* and *Calm2* are involved in regulating the hyperpolarizing effects of K^+^ channels;^40,41^ *NOS1AP* has been shown to cause NMDA receptor dysregulation associated with Schizophrenia;^42–44^ Arc increases the AMPA receptor endocytosis;^45–47^ *Rab27b*, *Lin7a* (previously known target^24^), Syt12 function relates to synaptic vesicle intracellular transport and exocytosis; and *Crh*, *Tac1* are neuropeptide and neurohormone. This upregulation of synapse genes may be due to the known role of Setd1a as modulator of Mef2c (Fig. 6b)^24^, which is a known activity-dependent transcriptional activator of synaptic genes during development^24,48^. The reduction in *Setd1a* expression level may lead to higher Mef2c-dependent transcription. To test this hypothesis, we compared the set of genes targeted by Mef2c, as identified by ChIP-seq experiments^24^, with upregulated and downregulated genes in *Setd1a*+/− MoNNets. Remarkably, we found 64 out of 100 upregulated genes in *Setd1a*+*/−* to overlap with known Mef2c targets (Fig. 6b, c). We performed bootstrapping to estimate the probability of the overlap just by random chance, and found it to be highly unlikely (P[overlap > = 64] ==1.0 × 10^−6^) as assessed by random sampling of 10^6^ sets of randomly chosen 100 genes from all expressed genes (Fig. 6d). In contrast, we found an overlap of only 18 out of 75 downregulated genes—much lower than expected by random chance (Fig. 6d). Therefore, the observed upregulation of synapse organization and signaling may be due to the reduced Setd1a-mediated modulation of Mef2c activity in *Setd1a*+/− MoNNets.

The downregulated genes in *Setd1a*+/− MoNNets relates to microtubule cytoskeleton, cell division, myelination, and neuronal survival (Fig. 6a). For example: Kif23, a molecular motor, has been shown to regulate the distribution of microtubules differentially into axons and dendrites in cultured rat superior cervical ganglia (SCG) neurons, thereby modulating the rate of axon versus dendrite outgrowth;^49–51^ Tpx, a microtubule-associated protein has been shown to be required for neurite elongation in mouse hippocampal neurons and rat SCG neurons;^52,53^ E2f1 and Chek1 are modulators of neuron survival and cell cycle;^54–56^ Bcas1, myelination associated protein, which has been linked with schizophrenia-like behavior abnormality^57^. Interestingly, consistent with these gene expression results, Setd1a -deficient neurons have been shown to have deficits in axon and dendrite growth and branching, as well as a reduction in mushroom and stubby spines in developing mouse cortical neurons in vivo as well as in vitro^24^.

Overall, the observed downregulation of cytoskeletal/connectivity and neuron survival genes, combined with upregulation of genes related to synapse organization and signaling, reveals the underlying molecular and cellular basis of the network-level global connectivity deficits observed in *Setd1a*+*/−* MoNNets. These results are in agreement with the structural connectivity, synaptic plasticity, and neuromodulation alterations associated with SCZ^18,19^.

### Partial pharmacological rescue of *Setd1a*+*/−* MoNNet network pathophysiology

**Fig. 7:**
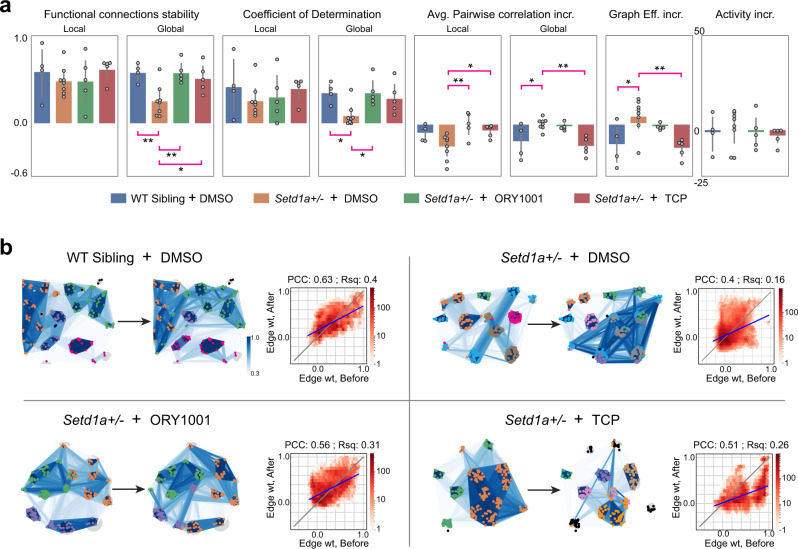
Partial rescue of *Setd1a*+*/−* MoNNet network pathophysiology by pharmacological inhibition of LSD1 demethylase activity. **a** Barplots comparing various quantitative measures of network function of WT sibling MoNNets (blue) and *Setd1a*+/− MoNNets treated with DMSO(orange), ORY-1001(green) and TCP(red). All treatments were for two days. From left to right: local and global functional connections stability as measured by the correlation in edge weights of functional graph before and after the treatment; local and global coefficient of determination (r-square fit edge weights before and after treatment) quantifying the predictability of the after-treatment state; average pairwise correlation increment as measured by increase in correlation of same pair before and after, averaged over an entire MoNNet; global graph efficiency increment measures of functional graphs; activity rate per minute. Statistical significance were calculated by using one-way ANOVA and Dunnett multiple comparison test with WT sibling or *Setd1a* + /− group as controls (***padj < 0.001; **padj < 0.01; *padj < 0.05). Error bars represent 95% confidence interval and center are means. All samples are biological replicates. **b**, Representative examples of WT sibling MoNNets and *Setd1a*+/− MoNNets treated with DMSO, ORY-1001, and TCP. Nodes belonging to same modules are colored same. Co-classification matrix heatmap and clustering dendrograms are shown to visualize the hierarchical relationship in modules activity. The density scatter plot compares edge weight before and after treatment. PCC: Pearson’s correlation coefficient of the scatter plot; Rsq: r-square measure of fitness of linear regression fit with a straight line. Source data are provided as a Source Data file.

To further assess the utility of SCZ-MoNNets as a quantitative phenotypic platform for drug discovery, we took advantage of recent findings indicating that LSD1 demethylase activity inhibitors, ORY-1001 and TCP, are effective in rescuing cognitive deficits when administered in adult *Setd1a*+*/−* mice^24^. However, the underlying network-level mechanism of action of these compounds is not known. As predictive experiments (in retrospect), we set out to test the effects of these inhibitors on MoNNets derived from *Setd1a*+*/−* and WT sibling controls at mature late culture ages (DIV 30–34). MoNNets from mutant and WT siblings were treated with ORY-1001, TCP and DMSO (vehicle) for two days. The neuronal activity was captured before and after the treatments, and the extracted activity sources were subjected to comparative analyses. As shown in Fig. 7, the treatment with these compounds resulted in a significant increase in functional connection stability as assessed by comparing the correlation in connection strengths (edge weights) of exact same pair of neurons before and after the treatment. Similarly, coefficient of determination, which quantifies predictability of the after-treatment network state, given the before state, was also increased. Furthermore, deviations in the pairwise activity correlations and graph efficiency were also recovered. Note that all these MoNNets are in transition periods, from phase II to phase III, with a reducing average pairwise correlation. The neuronal activity rates remained similar in all cases. Figure 7b shows representative examples of WT MoNNets, and SCZ-MoNNets treated with DMSO, ORY-1001, and TCP, visualizing the modular architecture and functional connection stability. These results strongly suggest that the inhibition of LSD1 demethylase activity in SCZ-MoNNets promotes stabilization of functional connections and modules.

## Discussion

Even though in vitro 3D neuronal culture approaches are reductionist in nature, they have the potential to allow the modeling of specific aspects of brain complexity to address well-defined questions. However, there are also potential limitations of in vitro systems in recapitulating certain in vivo physiological processes, including the existence of stress-induced abnormal gene expressions^58^. Most importantly, the current state-of-the-art approaches have remained largely incapable of capturing complex neural network properties of brain function. For example, a recent study^59^ successfully derived cerebral spheroids from 15 patients with *22q11.2* deletion syndrome to reveal alterations in the expression of genes related to neuronal excitability. However, these state-of-the-art spheroid preparations were not suitable for investigating the network function deficiencies because of their inherent limitations in recapitulating brain network properties. Recent developments in vascularization of organoids^60^ and assisted fusion of organoids from different brain regions^3,8^ may further improve their applicability by increasing the captured brain complexities, although, at a significant cost of much-enlarged sizes of these relatively opaque preparations.

Here we reported an alternate approach, so-called Modular Neuronal Networks (MoNNet), to address some of the limitations. The MoNNet provides several highly desirable features: (1) no practical limit on the size of the network, and hence the structural and functional complexity, while maintaining smaller accessible individual units, (2) systems-level experimental observation and manipulation access to individual neurons, modules, and the entire system, and (3) multi-scale complexity due to local and global modular connectivity patterns. Even though MoNNet approach does not capture developmental patterning to a similar extent as some of the organoids methods, their naturally hierarchic physical organization and the existence of mature neural circuits resembles some properties of the functional brain network architecture^17^. We tested the gene expression, cellular and functional properties of MoNNets, and found that indeed they can recapitulate aspects of brain networks, including mature neuronal networks, modularity, complex diversity of activity patterns, ensembles formation stability, interplay of excitation/inhibition balance, and segregated local-global synchrony.

Similar to developing embryos and standard organoids, MoNNet also exhibits significant sample-to-sample variations in the measurements of various neuronal and network properties. However, we found that the differential of after- and before- treatment measures of network properties in exact same sample, provide reliable quantitative measures of the effects of various stimulations (as shown in Figs. 2, 5, 7), thus demonstrating their utility as an effective approach for drug screening and for modeling of network dysfunction abnormalities associated with brain disorders. Note that we established and characterized MoNNet approach using murine cells, however, the long-term stability of MoNNet preparations (e.g. Supplementary Video 4 showing DIV 49 and DIV 64 samples) may be conducive to the future development of human iPSC derived MoNNet preparations in further studies.

Taking advantage of the MoNNet properties, we developed effective in vitro models of SCZ-associated network pathophysiology (SCZ-MoNNets), by building upon two well-studied genetic models—*Setd1a*+*/−* and *Df(16)A*+*/−* which recapitulate SCZ-related cognitive and circuitry pathophysiology^23,24^. An exhaustive systems-level comparative characterization of SCZ-MoNNets revealed degradation of modules/ensembles formation, altered global network synchrony, and much reduced inter-modular functional connections. In addition, RNAseq-based gene expression analysis of *Setd1a*+*/−* MoNNets revealed downregulation of cytoskeleton/neuronal structure-related genes and upregulation (via modulation of Mef2c activity) of synaptic signaling/function genes, revealing the underlying molecular mechanisms of observed network function deficits. Together with the regional in vivo observations^23,24^, these results provide network-level mechanistic insights into the causes of SCZ-associated behavioral abnormalities. The lower-level deviations in molecular, cellular, and synaptic pathways underlying the observed alterations in network states remain to be determined and may be different for each mutation. Nevertheless, it is worth noting that both mutations lead to alterations in terminal axonal growth, excitability and short-term plasticity while the *22q11.2* deletion has been shown to have an impact on inhibitory neuron function^23,24,32,61,62^. We also tested the applicability of SCZ-MoNNets as a potential drug screening system by characterizing the effects of antagonists of LSD1 methylase activity (ORY-1001 and TCP), which were recently shown to be effective in partial rescue of cellular and behavioral defects^24^. We found that even 2-day treatments of *Setd1a*+*/−* MoNNets with these compounds was sufficient to cause partial rescue of ensembles stability and network synchrony, revealing their mechanism of action. Even though a systematic screen for identifying novel psychoactive compounds was beyond the scope of this work, these experiments do demonstrate predictive power, retrospectively, for future screens of compounds with in vivo efficacy. Overall, this study provides a comprehensive network-wide cellular-resolution characterization of the pathophysiology caused by mutations predisposing to SCZ.

Altogether, these results strongly highlight the potential of MoNNet approach for more accurate in vitro representation of the network pathophysiology underlying complex brain disorders. By allowing systematic in vitro modeling of a variety of mutations linked to psychiatric and neurological disorders, this approach can facilitate detailed comparative characterization of the network dysfunction and its decomposition into common and divergent network features. MoNNets are very well suited for establishing high-throughput screens for systematic discovery of novel drug candidates.

## Methods

### Animals and genotyping

All animal handling and experimentations were done according to US National Institutes of Health guidelines and approved by the Institutional Animal Care and Use Committees (IACUC, AC-AABL2553) of Columbia University. Pregnant wild-type CD-1 mice were purchased from Charles River laboratories at E11.5, and maintained in a temperature-controlled environment on a 12-h light-dark cycle, with *ad libitum* food and water until the experiment day. *Df(16)A*^*+/−*^ and *Setd1a*^*+/−*^ mice were obtained from an in-house colony. Briefly, *Df(16)A*^*+/−*^ mice (RRID: MGI_3802827) were generated on a C57BL/6J background as described previously^25^. *Setd1a*^m1a(EUCOMM)Wtsi^ mice (referred to as *Setd1a*^*+/−*^) (https://www.infrafrontier.eu/search) backcrossed in the C57BL/6J (The Jackson Laboratory, Bar Harbor, ME) background^24^. Wild-type littermates from *Setd1a*^*+/−*^ crosses to WT mice were used as controls. The embryos were genotyped by PCR analysis of tail genomic DNA. For *Setd1a* crosses, following primer combinations were used: Setd1a_F (5′-GGTTATTGATCTGGGCAGGC-3′) and Setd1a_R (5′-TGACCTGTTTTTCAAGCCCTC-3′); Setd1a_F and CAS_R1_Term (5′-TCGTGGTATCGTTATGCGCC-3′), to amplify the wild-type and mutant alleles with 546 and 241 base pairs expected band sizes respectively. For *Df(16)A*^*+/−*^ crosses, Df(16)A_F (5′-ATTCCCCATGGACTAATTATGGACAGG-3′) and Df(16)A_R (5′-GGTATCTCCATAAGACAGAATGCTATGC-3′) were used to amplify a 829 bp band for mutant allele, and Control_F (5′-CTAGGCCACAGAATTGAAAGATCT-3′) and Control_R (5′-GTAGGTGGAAATTCTAGCATCATCC-3′) were used to amplify a 324 bp IL-2 internal control band. The PCR program comprised denaturation at 94 °C for 5 min, followed by 35 cycles of 30 s at 94 °C, 30 s at 58 °C, and 45 s at 72 °C, and a final extension step at 72 °C for 5 min. For *Thy1-eYFP* transgenic lines experiments, *B6.Cg-Tg(Thy1-YFP)HJrs/J* strain mice were procured from Jackson Laboratory (Ref. 003782), and bred in Columbia University animal facilities. Embryos were genotyped by PCR analysis of the tail genomic DNA using 5′ CGGTGGTGCAGATGAACTT 3′ and 5′ ACAGACACACACCCAGGACA 3′ primers. PCR program comprised denaturation at 94 °C for 2 min, followed by 35 cycles of 20 s at 94 °C, 15 s at 65 °C, and 10 s at 68 °C, and a final extension step at 72 °C for 2 min.

### 3D Cell-culture preparation

Hippocampal neuronal cultures were generated from E17 to E18 embryos by building upon standard cell-culture techniques, as follows. Pregnant mice were anesthetized under isoflurane, and euthanized by cervical dislocation. Hippocampus was dissected in Hibernate E (Gibco) iced cold media, and incubated in 0.25% Trypsin-EDTA (Gibco) at 37 °C for 30 min., followed by 5 min DNAse I (1 μg/ ml; Sigma) incubation at room temperature. Mechanical dissociation of dissected hippocampus was performed by repeated pipetting with a fire-polished glass Pasteur pipet until a homogenous cell suspension was obtained. Note that, for preparing MoNNets from CD1 mice, the dissociated cells from all pups in a given litter were mixed, whereas for MoNNets from *Setd1a*+*/*, *Df(16)A*^*+/−*^ and their WT siblings, cells from each embryo were processed separately to generate MoNNets. Cell viability was determined by Trypan Blue exclusion assay. The cell solution was then centrifuged at 150*g* for 10 min, and the supernatant was removed. The resulting cell pellet was resuspended in the culture media containing Neurobasal media, 2% B27, 0.5 mM Glutamate and 1% Penicillin/Streptomycin (Gibco). Cells were infected with AAV1.Syn.GCaMP6f.WPRE.SV40 virus (^63^; Pennsylvania Vector Core, Cat#:AV-1-PV2822) for neuronal GCaMP6f expression. The viral infection at single-cell suspension stage, just before seeding, allowed for infection of most cells. It is well documented that following similar procedures, AAV1 is transduced in over 94.1% of all cells with approximately 80% of them being neurons^64^. For single isolated spheroid cultures, 2% agarose 96 wells (400 μm diameter) micro-molds were created by using custom casts fabricated by 3D printing (UV-resin; Formlabs 3D printer) as follows: 2% agarose and 1% sucrose in water was microwaved to completely dissolve; the mixture was poured into the 3D-printed cast and solidified at room temperature; and the resulting agarose micro-molds were equilibrated in culture media for at least 24 h. Approximately 10^5^ cells were seeded in the agarose micro mold. For the MoNNet samples, we generated custom polydimethylsiloxane(PDMS) molds, containing four 28 mm diameter wells, as follows: (1) custom cast were 3D-printed with acrylonitrile butadiene styrene (ABS; Ultimaker 2+), (2) fresh PDMS was prepared my mixing uniformly the silicone elastomer base and the curing agent at a ratio of 10:1 (weight/weight), followed by degassing in a vacuum chamber, (3) the freshly prepared PDMS was poured on to the cast and covered with a coverslip, (4) PDMS was cured at 90 °C overnight, (5) the resulting PDMS molds were sterilized by immersing in 100% alcohol overnight. Approximately 2 × 10^5^ cells were seeded in each of the four wells in the PDMS mold. For both individual isolated spheroids and MoNNets cultures, the cells were allowed to settle for 15 min, followed by addition of 2 ml of culture media. All cell cultures were kept in an incubator at 37 °C and 5% CO_2_. Immunostainings with Caspase-3 were used to assess for any apoptosis in the preparations.

### Validation of MoNNets

Extensive validation was performed at each stage of MoNNet preparations. (1) In the early steps of MoNNet preparations, Trypan Blue exclusion assays were performed to ensure the viability and counts of the cells that were used for seeding of the cultures. (2) Caspase-3 immunostainings were performed to tests for any significant cell deaths. A representative example is shown in Supplementary Fig. 5. (3) Morphology of all MoNNet preparations was closely inspected for any damages and debris. (4) Existence of neuronal activity also provided indications for the cultural health. For example, Supplementary Video 4 shows the activity of several months old (DIV 49 and DIV 64). (5) Embryos were genotyped by PCR analysis of the tail genomic DNA, as detailed above.

### Total RNA preparation and sequencing

MoNNet and spheroid preparations were first washed with 3x RNase-free phosphate-buffer saline. 800 μl QIAzol lysis reagent (Qiagen, Cat#79306) was added for 5 min at room temperature, followed by the addition of 200 μl Chloroform (Fisher Chemical, Cat#C298–500), vigorous shaking for 15–30 s, incubation for 3 min at RT, and centrifugation (12,000*g*) at 4 °C. The aqueous upper phase was collected to a new tube, 500ul Isopropanol (Sigma, Cat#I9516–500ml) was added, vortexing was used for mixing. After 10 min incubation at RT, the solution was centrifuged (12,000*g*) for 10 min at 4 °C. The supernatant was removed to collect the pellet, which was briefly air-dried. 100ul RNase-free water was used to dissolve the pellet. RNeasy Mini kit50 (Qiagen, cat#74104) was used to clean the RNA - all steps performed at 4 °C. RNA concentrations were measured by Nanodrop 2000 spectrophotometer (Thermo Scientific) and bioanalyzer was used for RNA quality assessment. Poly-A pull-down was used to enrich mRNAs from total RNA samples, followed by library construction using Illumina TruSeq chemistry. Libraries were sequenced on the Illumina NovaSeq 6000 at the Columbia Genome Center, yielding ~20 million paired-end 100 bp reads for each sample. RTA (Illumina) for base calling and bcl2fastq2 (version 2.19) for converting BCL to fastq format, coupled with adaptor trimming.

### Pharmacology

Synaptic and ion channel inhibition experiments were performed with bicuculline (10 μM, Cat# 14340, Sigma), picrotoxin (10 μM, Cat# P1675, Sigma), NBQX (10 μM, Cat# N183, Sigma), D-APV (40 μM, Cat# A8054, Sigma), Nifedipine (10 μM, Cat# N7634, Sigma), TTX (1 μM, Cat# 1078, Tocris) and Mefloquine (25 μM, Cat# M2319, Sigma). The rescue experiments were performed with Tranylcypromine (TCP, 600 nM, Sigma, P8511) and ORY-1001 (0.6–0.9 nM, Cayman Chemical, 19136). All the compounds were added to the culture media as described in the results.

### Time-lapse imaging

Most of the imaging experiments were performed using a wide-field fluorescence microscope (Leica M165FC) equipped with a long-pass GFP filter set (Leica filter set ET GFP M205FA/M165FC), 1.6x Plan Apo objective, 3.2x zoom and sCMOS camera (Hamamatsu ORCA-Flash 4.0). Micromanager (v1.4.22) and HCImage (v4.5) software were used for image acquisition. Time-lapse videos were recorded at 30 Hz for ~4.5 min at 37 °C in culture media (Neurobasal media, 2% B27, 0.5 mM Glutamate and 1% Penicillin/Streptomycin (Gibco) with petri dish ring heater (TC-E35x15) and programable heating controller (TC2-80-150/BTC-2-100; Bioscience Tools) to maintain temperature at 37 °C. No 5% CO_2_ gas equilibrium was used for these short recordings.

### Immunohistochemistry

MoNNet samples were fixed for 30 min at 4 °C in 4% PFA, and immunostaining was performed on either vibratome sections (50 µm) or whole mount MoNNets. For vibratome section staining, the sections were washed in PBS + 0.1% Triton X-100 and incubation in blocking solution (PBS + 0.1% Triton X-100 + 1% BSA) for 40 min, followed by overnight incubation with primary antibody (in blocking solution) at room temperature. Then sections were washed with PBS–0.1% Triton X-100, followed by incubation in secondary antibodies(1:500 dilution, in blocking solution) for 2 h. Finally, sections were washed with PBS–0.1% Triton X-100 and mounted on slides for Confocal imaging. The following antibodies were used: Rabbit α-Glutaminase (1:500, Cat# Gltn-Rb-Af340, Frontier Institute Co. LTD.), mouse α-NeuN, clone A60 (1:50, Cat# MAB377, Millipore), mouse α-GAD65 (1:1000, GAD-6, DSHB), rabbit α-GFAP (1:500, Cat# Z0334, Dako), rat α-phospho-Histone H3 (1:200, Cat# h9908, Sigma), mouse α-TUJ1 (1:200, Cat# 801201, BioLegend) and rabbit α-Caspase3(1:500, Cat# 559565, BD Pharmingen). Alexa Fluor 568-, and 647- conjugated secondary antibodies (goat anti rabbit 647 cat# A21245, goat anti Rabbit 568 cat# A11011, goat anti Mouse 647 cat# A21235, goat anti Mouse IgG2a 647 cat#A21241, Invitrogen; 1:500 dilution) were obtained from Invitrogen. For whole mount MoNNets, the antibody blocking solution consisted of PBS + 0.3% Triton X-100 + 0.5% BSA. Primary antibodies were incubated for 3 days at 4^o^C while the secondary antibody for 4 h at room temperature. DAPI was used at 1 µg/ml, and used with the secondary antibody. Images were acquired using confocal microscope (Zeiss, LSM700 with Zeiss software) using 10x(EC Plan-Neofluar 10x/0.30 M27) and 20x(HC Plan Apochromat, NA 0.70).

### Electrophysiology

Whole-cell recordings were performed as described previously^65,66^, using borosilicate glass pipettes (initial resistance 3–5.5 MΩ) which were filled with an intracellular solution containing: K methanesulfonate 125 mM, NaCl 10 mM, CaCl_2_ 1 mM, MgCl_2_ 1 mM, HEPES 10 mM, EGTA 0.1 mM, MgATP 5 mM, NaGTP 0.5 mM. The pH was adjusted to 7.2 with KOH. Spontaneous synaptic events were assessed at −70 mV (presumptive glutamatergic) and at 0 mV (presumptive GABAergic). Excitatory and inhibitory evoked synaptic responses were assessed as follows. Evoked synaptic responses across spheroid units were elicited with electric stimulation applied with a concentric bipolar stimulating electrode (tip diameter 0.125 mm, FHC, Bowdinham, ME) positioned on a spheroid >250μm away from the spheroid containing the recorded neuron. The stimulus was set to 10 V, a duration of 100μs and applied at 10 Hz. After achieving whole cell configuration, neurons were initially held in voltage-clamp at −70 mV to assess evoked excitatory synaptic responses, followed by washing-in of CNQX (10 μM) with ACSF (artificial cerebral spinal fluid) perfusion which completely ablated evoked excitatory synaptic responses at −70 mV, confirming these responses as mediated by glutamate. Neurons were then held in voltage clamp at 0 mV to assess evoked inhibitory synaptic responses. For assessing neuronal excitability in current step, action potential firing was recorded in response to incremental (20pA steps) depolarizing current injections (500 ms duration). Bridge balance of series resistance was employed and recordings with series resistance > 20 MΩ were rejected. For current-step assays, resting membrane potential was adjusted to ~ −70 mV by injection of a small standing current. For voltage steps, cells were held at −70 mV, and 10 mV steps were applied ranging from −100mV to 50 mV. Series resistance-related errors were partially corrected by using 70% prediction and 70% series resistance compensation.

### RNAseq data analysis

kallisto^67^ (0.44.0,R package) was used to perform pseudo-alignment with a kallisto index created from mouse transcriptome (GRCm38), followed by conversion to gene counts by tximport package. DESeq2^68^ (R package) was used for differential gene expression analysis among different samples. The batch variability of different sequencing runs was accounted for by defining “batch” as a covariate in the linear model to analyze differential gene expression. Differential gene expression heat maps were generated by using pheatmap (v1.0.12, R package), and volcano plots by EnhancedVolcano (v1.10.0, R package). Gene ontology (GO) enrichment analysis was performed by using topGO^37^ (v2.44.0, R) and ViSEAGO^38^ (v1.6.0) package with “elim” algorithm and *p*-value cutoff of 0.01. R (v 4.1.1) was used for all the analysis.

### Functional imaging data analysis

#### Motion artifacts correction

All Ca^2+^ imaging datasets were first manually screened for any motion artifacts during the recordings. Datasets having motion artifacts were corrected by using the MOCO algorithm implemented as plugin^69^ in ImageJ/Fiji (v1.53c)^70,71^.

#### Segmentation and aggregated signal of Spheroid units

A custom watershed segmentation pipeline was implemented in Python by using the scikit-image module^72^. Maximum intensity projection images (along the time axis) were used as inputs for segmenting the individual spheroid units. The resulting segmentation labels were manually inspected for any errors, which were corrected by adjusting the upper and lower thresholds, and the object size filter. These segmented images were also used for estimating the size distributions and distances among the nearest neighbors (geometric center to geometric center). Aggregated signal traces of each spheroid unit were calculated as average values in labels overlaid on Ca^2+^ imaging background-subtracted time-frames. Note that the background of each time frame was estimated as the average pixel value outside the total segmentation mask. The aggregated signal was normalized by subtracting and dividing by the baseline values (estimated as the 8th percentile value in a sliding window of 500 frames).

#### Neuronal activity traces extraction

A custom pipeline in Python (using scientific modules numpy v1.190, scipy v1.6.0, Scikit-image v0.15) was used to localize activity sources by performing joint spatio-temporal deconvolution using constrained non-negative matrix factorization (CNMF)^73–75^. Spurious non-spheroid sources were filtered by using the spheroids segmentation masks. For each identified unique spatial footprint, top 25% most probable pixels were used to calculate the average signal from background-subtracted images. Signal traces were normalized (∂F/F) by subtracting and dividing by the baseline values, which was estimated as 8th percentile signal in a sliding window of 500 frames. A medium filter was applied to denoise the traces. Activation traces were temporarily deconvolved to infer activity onsets using OASIS method^76^. Second order generative autoregression model was used.

#### Local and global average pairwise calculations

Average pairwise activity correlation was calculated as the average of Pearson’s correlation coefficient in ∂F/F traces of all possible pairs of neurons in a sample. The local and global average pairwise correlation was calculated only from the pairs belonging to the same and the different spheroid units, respectively.

#### Activity rate and clustered activity duration

Activity-onset rate (per minute) was calculated from estimated activity-onset trains. A threshold of 5 standard deviations was used to identify significant activity-onset events for calculations. Average activity rates were calculated by averaging over all neuronal sources belonging to a MoNNet sample. Clustered activity duration was calculated as the full width at the level of 75% or 50% of the ∂F/F peak, as follows. For the first identified activity-onset event in the time series, a forward iterative traversal was performed on ∂F/F time series to identify the peak time position, followed by forward and backward iterative traversal to calculate the full width at 75% (or 50%) of the peak level relative to the baseline. The procedure was repeated for the next activity-onset position, not overlapping with the already analyzed peaks. The average value was calculated for all peaks identified in an individual neuron, which were then averaged over all neurons to yield the average clustered activity duration.

#### Local and global graph efficiency calculations

In the first step, weighted graphs were generated by representing neurons as nodes, and the pairwise correlations between a pair of neurons as the weighted edge. A threshold of 0.8 was consistently applied on the edge weights to generate binary graphs, which were used to calculate the global and local graph efficiencies using the NetworkX (v2.4) python modules. Note that the trends looked similar for a range of weight thresholds.

#### Modularity and co-classification analysis

Louvain community detection algorithm^35^ was used to detect modules in the MoNNet weighted graphs (discussed above). Note that a lower threshold of 0.5 was used to remove the weaker edges from the weighted graphs for these calculations. Nodes co-classification (into the same modules) probabilities were calculated by using multiresolution modularity and consensus clustering^36^ analysis of binary graphs generated by consistently using an edge weight cutoff of 0.7. This method (MATLAB (R2020b) implementation) utilizes multiresolution modularity function (Reichart and Bornholdt^77^, with γ as the resolution parameter) to generate an ensemble of community partitions of the graph at different scales, by uniform coverage of the entire range of the resolution parameter (γ, see^36^ for details of ad hoc determination of γ min-max range and the sampling method). These ensembles of community partitions are then used to calculate the co-classification (into the same module) probabilities for all pairs of nodes across scale, resulting in the final hierarchical consensus community structure. We used 10,000 uniform sampling (the only input parameter of the procedure^36^) of the range of modularity resolution parameter (γ) to achieve good coverage of the complete possible scaling of networks.

#### Activity source extractions from before and after treatment MoNNets

Ca^2+^ imaging datasets before and after pharmacological treatments were first aligned using a custom registration pipeline based on SimpleITK python implementation^78^. Maximum projection(along time axis) images from after-treatment datasets were registered to the corresponding maximum projection images from before-treatment datasets. Translational transformations and Mattes’ mutual information similarity matrix^79^ were used for performing the registration. Optimized translational parameters were then applied to the entire after-treatment image stacks, to yield completely aligned datasets, which were inspected manually to ensure high cellular-resolution accuracy. Finally, before and after treatment stacks were concatenated to detect activity sources using the pipeline discussed above, followed by the calculation of various measures discussed above.

### Reporting summary

Further information on research design is available in the Nature Research Reporting Summary linked to this article.

## Supplementary information

Supplementary Information

Description to Additional Supplementary Information

Supplementary Video 1

Supplementary Video 2

Supplementary Video 3

Supplementary Video 4

Supplementary Video 5

Supplementary Video 6

Supplementary Video 7

Supplementary Video 8

Reporting Summary

## Data Availability

All data are available from authors upon request. Bulk RNA sequencing data are available under BioProject accession code PRJNA832747. Publicly available Mef2 data used is available under accession code GEO: GSE123652 (Mukai, J. et al. Neuron, doi:10.1016/j.neuron.2019.09.014 (2019)). Source data are provided with this paper.

## Source data

Source Data

## Code availability

Custom analysis code used is available at https://github.com/tomerlab/Rabadan_Tomer2022 and upon request.
