# Supplementary material for: An in vitro model of neuronal ensembles
Source: Nat Commun. 2022 Jun 9;13:3340. doi: 10.1038/s41467-022-31073-1 (PMC9184643; doi:10.1038/s41467-022-31073-1)
Supplement: Supplementary file 1 — Supplementary Information [file 41467_2022_31073_MOESM1_ESM.docx]

**Supplementary information**

**An *in vitro* model of neuronal ensembles**

M Angeles Rabadan^1^, Estanislao Daniel De La Cruz^1^, Sneha B. Rao^2^, Yannan Chen^1,3^, Cheng Gong^1,3^, Gregg Crabtree^2^, Bin Xu^4^, Sander Markx^4^, Joseph A. Gogos^2,5,6,7^, Rafael Yuste^1,8^, Raju Tomer^1,2,3,8,*^

*Corresponding author: raju.tomer@columbia.edu

**Affiliations**

^1^Department of Biological Sciences, Columbia University, New York, NY, USA.

^2^Mortimer B. Zuckerman Mind Brain and Behavior Institute, Columbia University, New York, NY, USA.

^3^Department of Biomedical Engineering, Columbia University, New York, NY, USA.

^4^Department of Psychiatry, Vagelos College of Physicians & Surgeons, Columbia University, New York, NY, USA.

^5^Department of Physiology, Columbia University, New York, NY, USA.

^6^Department of Neuroscience, Columbia University, New York, NY, USA.

^7^Department of Psychiatry, Columbia University, New York, NY, USA.

^8^NeuroTechnology Center, Columbia University, New York, NY, USA.

**Table of contents**

**Supplementary Fig. 1** | Representative raster plots of individual spheroids and Modular Neuronal Network (MoNNet)

**Supplementary Fig. 2** | Three phases of MoNNets maturation

**Supplementary Fig. 3** | Representative raster plots of phase I MoNNet activity before and after 1 hour of pharmacological treatments with synaptic and ion channels inhibitors

**Supplementary Fig. 4** | Representative examples of co-classification matrix for different graph edge weight thresholds

**Supplementary Fig. 5** | Representative raster plots of phase II MoNNet activity before and after 1 hour of pharmacological treatments with synaptic and ion channels inhibitors

**Supplementary Fig. 6** | Dose-dependent effects of Bicuculine and Picrotoxin on MoNNets activity and synchronization

**Supplementary Fig. 7** | Representative examples of GAD65 whole-mount immunostaining of 4 weeks old MoNNets

**Supplementary Fig. 8** | Immunolabeling of spheroid sections and electrophysiological recordings of spontaneous action potentials

**Supplementary Fig. 9** | Gene expression heat maps for down-regulated genes in MoNNets at 30 DIV, relative to 15 DIV

**Supplementary Fig. 10** | Comparative enrichment of Gene Ontology: Biological Process terms in up (green) and down (blue) regulated genes in MoNNets, relative to spheroids

**Supplementary Fig. 11** | Comparison of MoNNets derived from WT CD-1 and WT C57BL/6J.

**Supplementary Fig. 12** | Comparative enrichment of Gene Ontology: Biological Process and Cellular Component terms in up (green) and down (blue) regulated genes in Setd1a +/-, relative to WT siblings.

**Supplementary Video 1** | Representative calcium imaging data of individual spheroids and highly interconnected MoNNet

**Supplementary Video 2** | Representative calcium imaging data of MoNNets in three phases

**Supplementary Video 3** | Representative calcium imaging data of isolated spheroids in three phases

**Supplementary Video 4** | Representative calcium imaging data of older MoNNet samples

**Supplementary Video 5** | Representative calcium imaging data before and after Bicuculine [10μM] treatment

**Supplementary Video 6** | Representative calcium imaging data of controls for the pharmacological treatments

**Supplementary Video 7** | Confocal z-stack movie visualizing the 3D cellular architecture of MoNNet

**Supplementary Video 8** | Representative calcium imaging data from WT, Setd1a+/- and Df(16)A+/- MoNNets


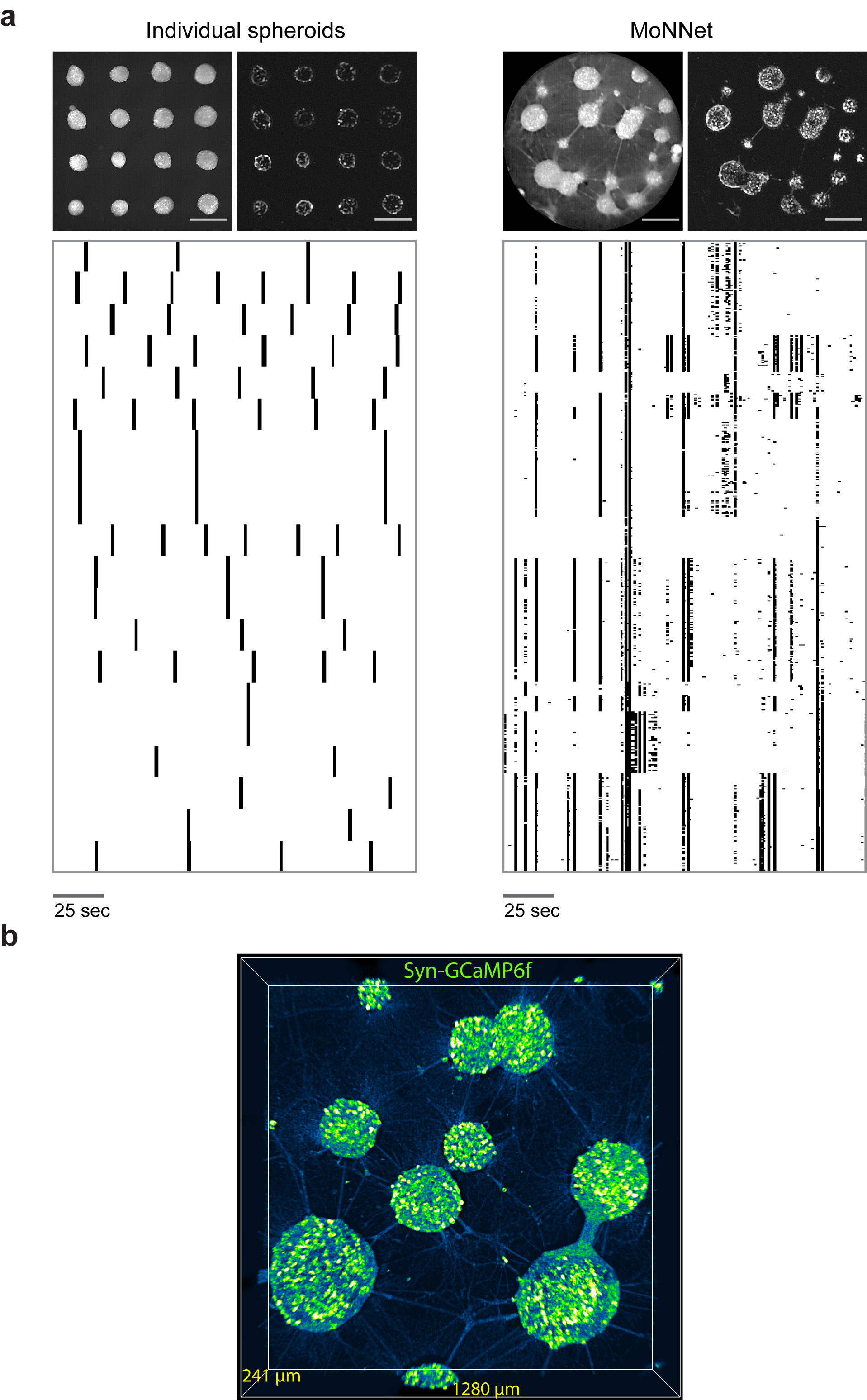


**Supplementary Fig. 1. Representative raster plots of individual spheroids and Modular Neuronal Network (MoNNet). a,** Left to right: Neuronal activity raster plots extracted from spheroid and MoNNet. Images shown are maximum projection across time and corresponding intermediate peak signal-to-noise ratio images from the CaImAn based analysis pipeline for activity source extraction. The neurons belonging to same spheroids are grouped together in raster plots. Scale bars are 500 μm. These representative experiments were independently observed >10 times. **b**, Volume rendering of baseline GCaMP6f signal in a fixed 4 weeks old MoNNet. These representative images of GCaMP expression were independently observed in live imaging of >200 samples as described in Fig 1.


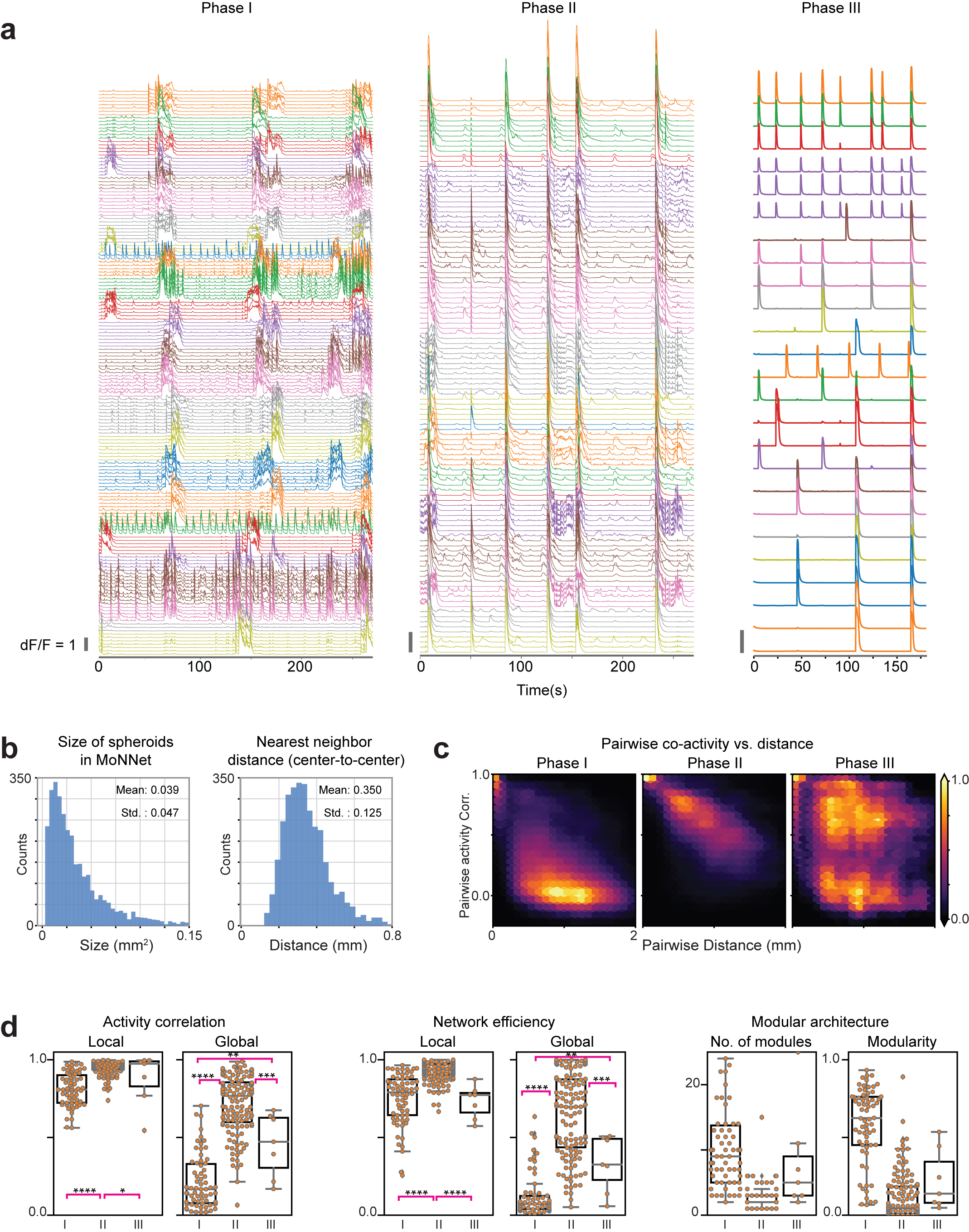


**Supplementary Fig. 2. Three phases of MoNNets maturation. a**, Neuronal activity traces extracted from MoNNets in three phases of network activity. Neurons belonging to same spheroids are grouped together in same color. **b**, Distribution of spheroid sizes in MoNNets, calculated from MoNNets of CD1 background and distances with their nearest neighbor, geometric center to geometric center. **c**, Pairwise activity correlation vs. pairwise distances in three phases. **d**, Left-to-right: Comparison of the pooled data in three phases: local (green) and global (blue) average pairwise correlation; local (green) and global (blue) network efficiency of weighted functional graphs; number of detected modules; modularity measure Q. n (biologically independent samples) for phase I = 66, phase II = 130 and phase III = 7. Statistical significance was calculated by one-way ANOVA with post-hoc Tukey’s test for multiple comparisons: * padj<0.05, **padj<0.01, ***padj<0.001, ****padj<0.0001. Note that the global activity correlation from **Fig. 1d** is plotted again for completeness. The boxplots show minimum, first quartile, median and third quartile. Source data are provided as a Source Data file.


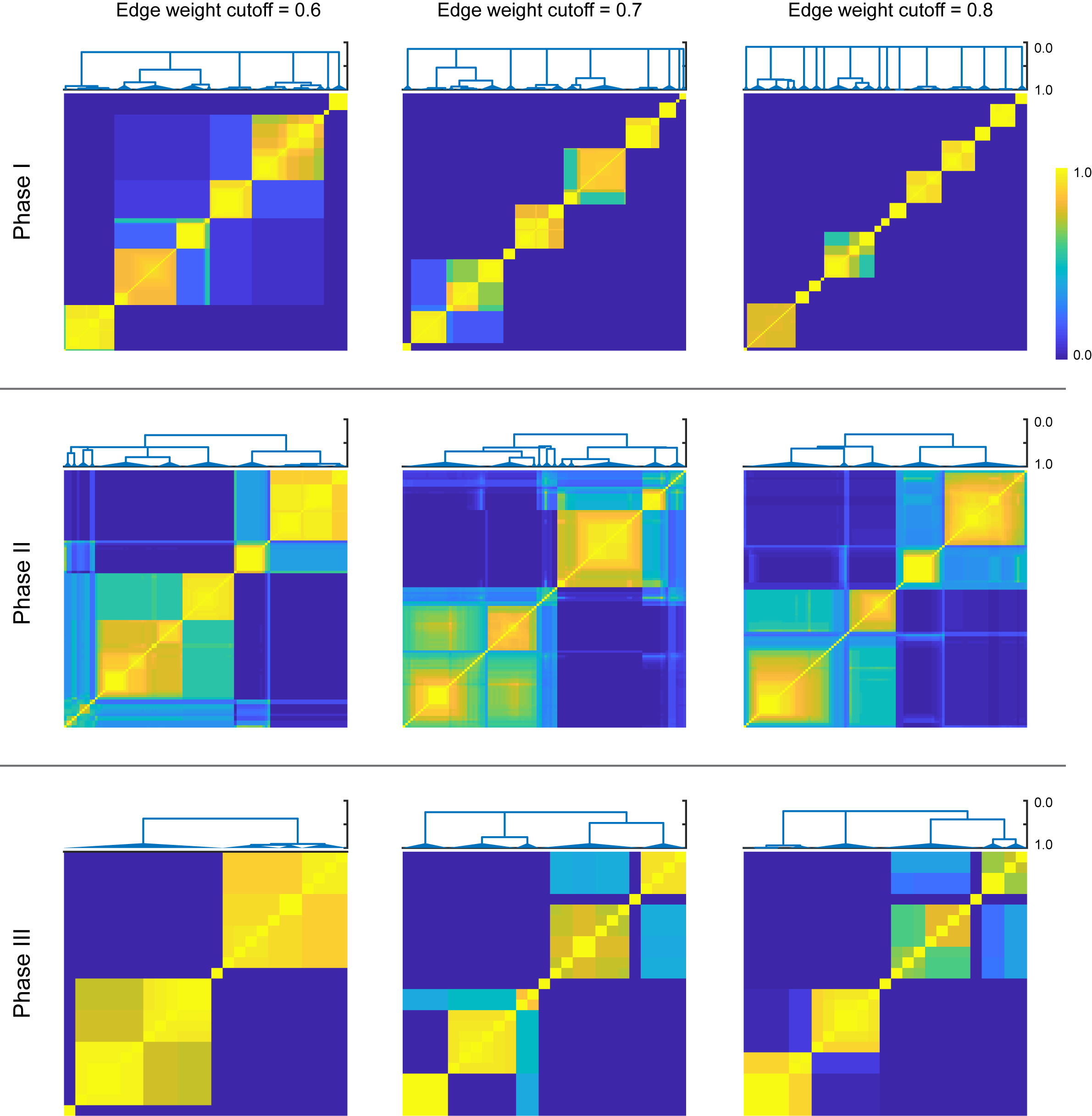


**Supplementary Fig. 3. Representative examples of co-classification matrix for different graph edge weight thresholds.**


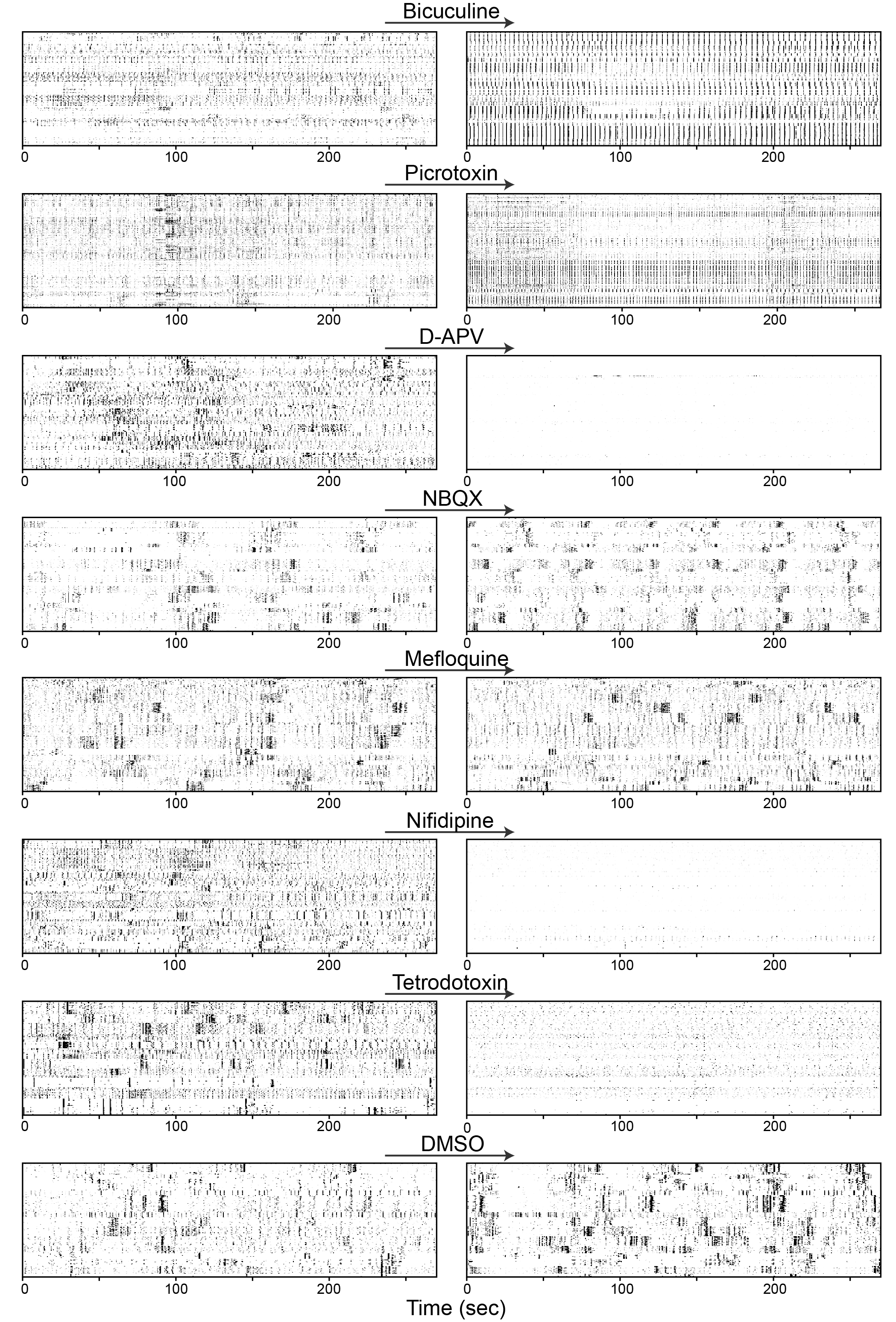


**Supplementary Fig. 4. Representative raster plots of phase I MoNNet activity before and after 1 hour of pharmacological treatments with synaptic and ion channels inhibitors.**


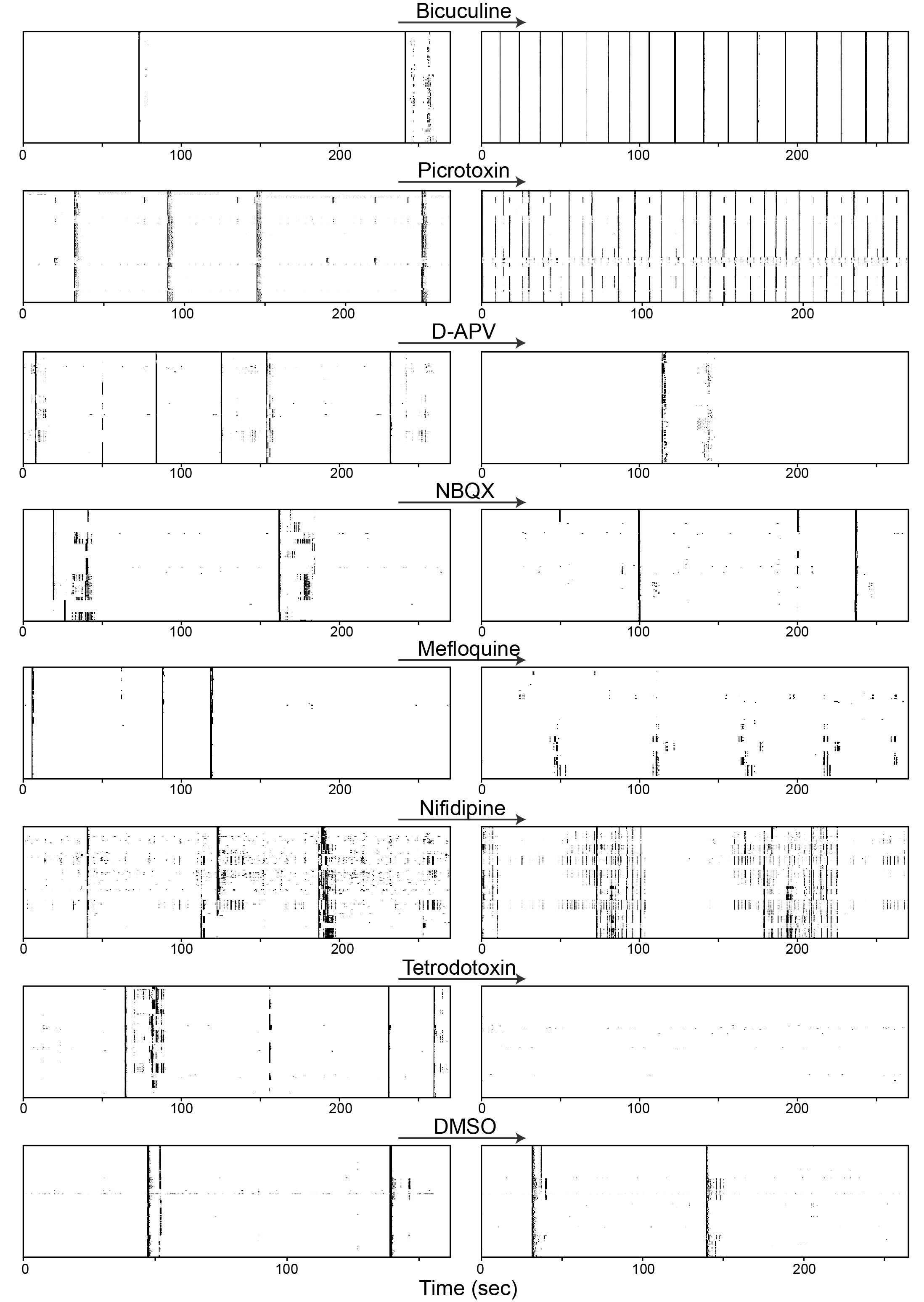


**Supplementary Fig. 5. Representative raster plots of phase II MoNNet activity before and after 1 hour of pharmacological treatments with synaptic and ion channels inhibitors.**


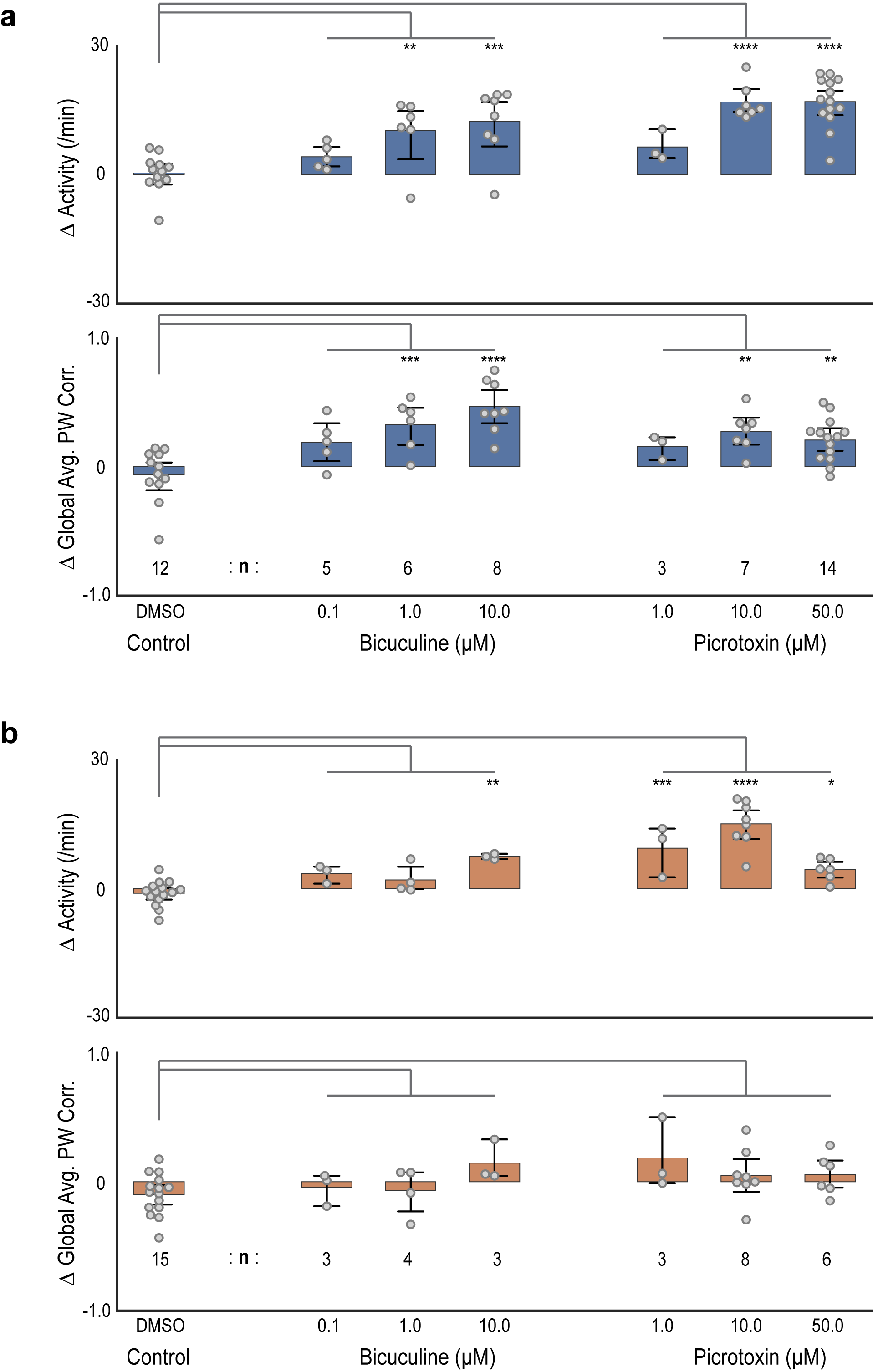


**Supplementary Fig. 6. Dose-dependent effects of Bicuculine and Picrotoxin on MoNNets activity and synchronization.** MoNNets in phase I (a) and phase II (b) were treated with multiple concentrations of GABA receptor antagonists Bicuculine and Picrotoxin. The Y-axes quantifies the increments in activity and global co-activity after 1 hour of treatments. Statistical significance was calculated by using one-way ANOVA and Dunnett multiple comparison test. * padj<0.05, **padj<0.01, ***padj<0.001, ****padj<0.0001. The error bars are 95% confidence interval. Source data are provided as a Source Data file.


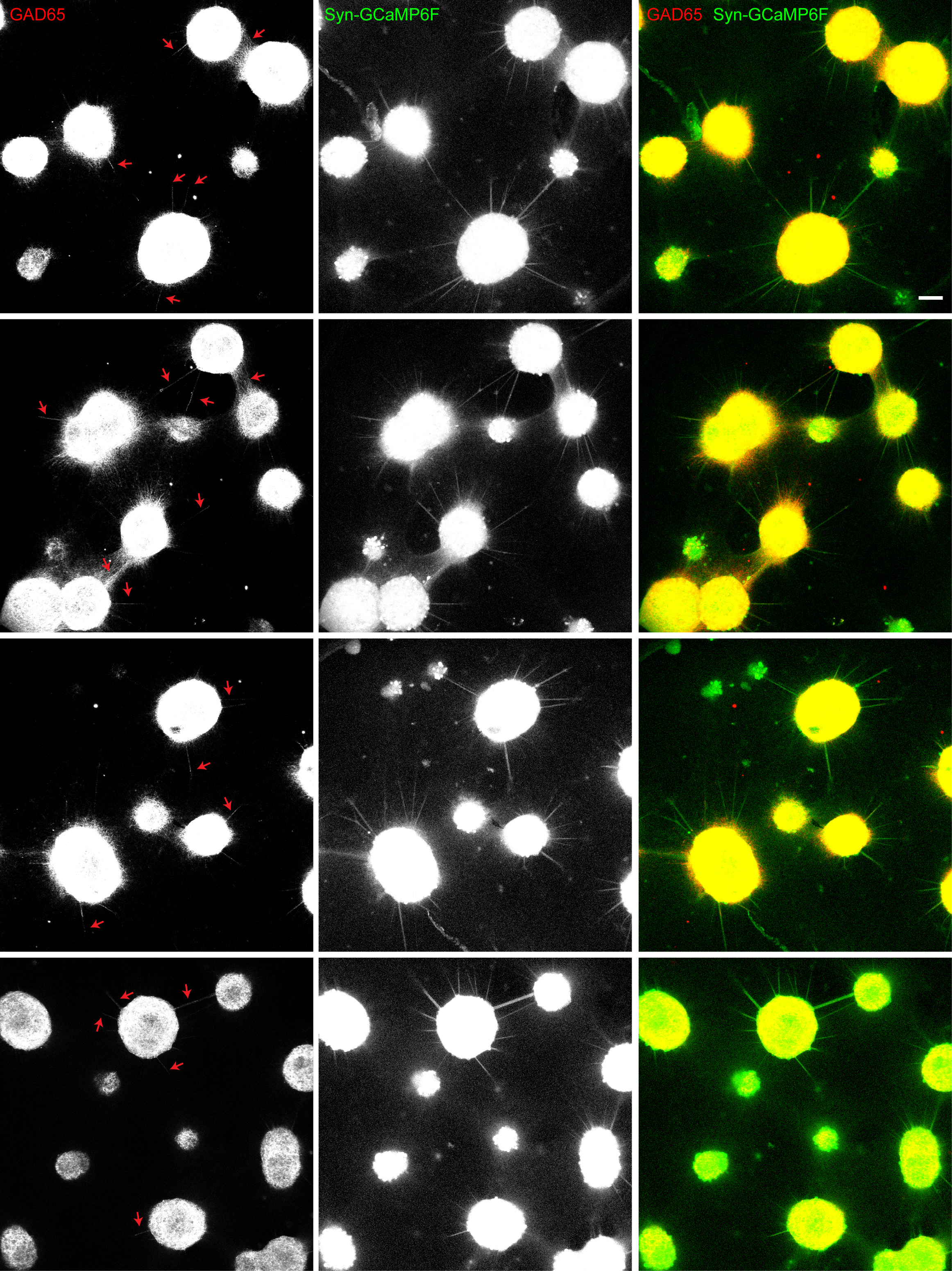


**Supplementary Fig. 7. Representative examples of GAD65 whole-mount immunostaining of 4 weeks old MoNNets.** Scale bar is 100 µm. Images from 4 independent samples shown. The staining experiments were repeated 3 times.


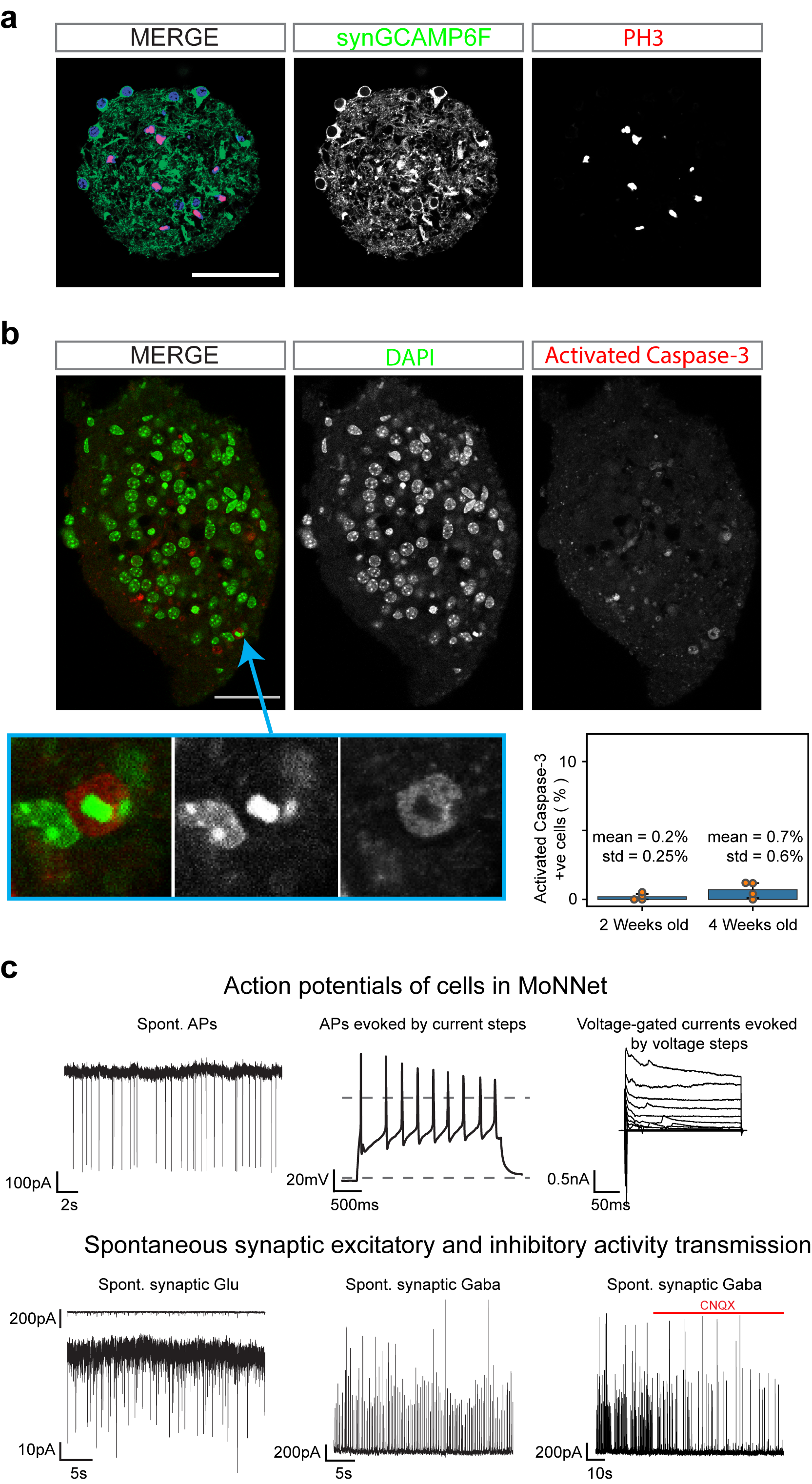


**Supplementary Fig. 8. Immunolabeling of spheroid sections and electrophysiological recordings of spontaneous action potentials.** **a-b**, Immunolabelling of PH3 and activated Caspase-3 demonstrates existence of proliferating cells, and general absence of cell deaths Quantification of activated caspase-3 labelling was performed on images from n=4 2-weeks old and n=4 4-weeks old MoNNets samples. Representative images of DAPI (green) and activated Caspase-3 (red) staining of 4-week old MoNNet sample are shown. Scale bars are 100 (a) and 50 µm (b). The staining experiments were repeated 3 times. Error bars represent 95% confidence interval and center points are mean. **c,** Top, left-to-right: Representative trace of a cell-attached recording from MoNNet (DIV14-15), showing spontaneous action potential (AP) at ~1.5Hz; representative traces showing robust AP responses to 400pA depolarizing current steps (reference lines: -70mV and 0mV levels); representative traces of voltage-step (-100mV to 50mV, cells held at -70mV between voltage steps) evoked currents showing robust voltage-gated sodium currents and voltage-gated potassium channel currents. Bottom, left-to-right: representative 30s trace of a neuron held at -70mV showing high frequency, small amplitude excitatory synaptic responses; representative 30s trace of a neuron held at 0mV showing very large, high frequency (> 3Hz) spontaneous inhibitory synaptic events; representative 100s trace of a neuron held at 0mV showing large, high frequency spontaneous inhibitory synaptic events and its reduction in presence of CNQX (10 μM). Source data are provided as a Source Data file.


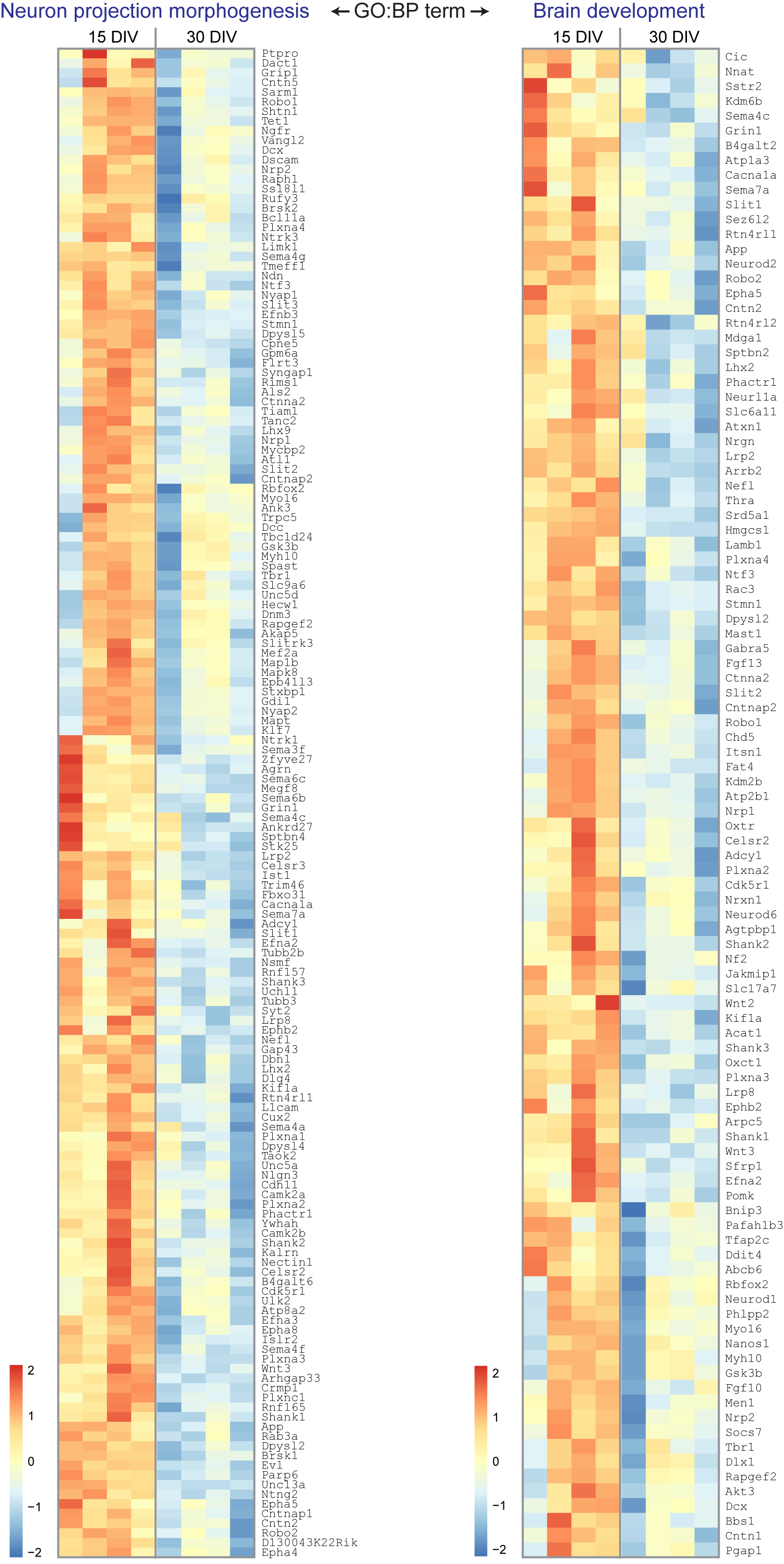


**Supplementary Fig. 9. Gene expression heat maps for down-regulated genes in MoNNets at 30 DIV, relative to 15 DIV.** Down-regulated genes associated with GO:BP terms “Brain development” and “Neuron projection morphogenesis”.


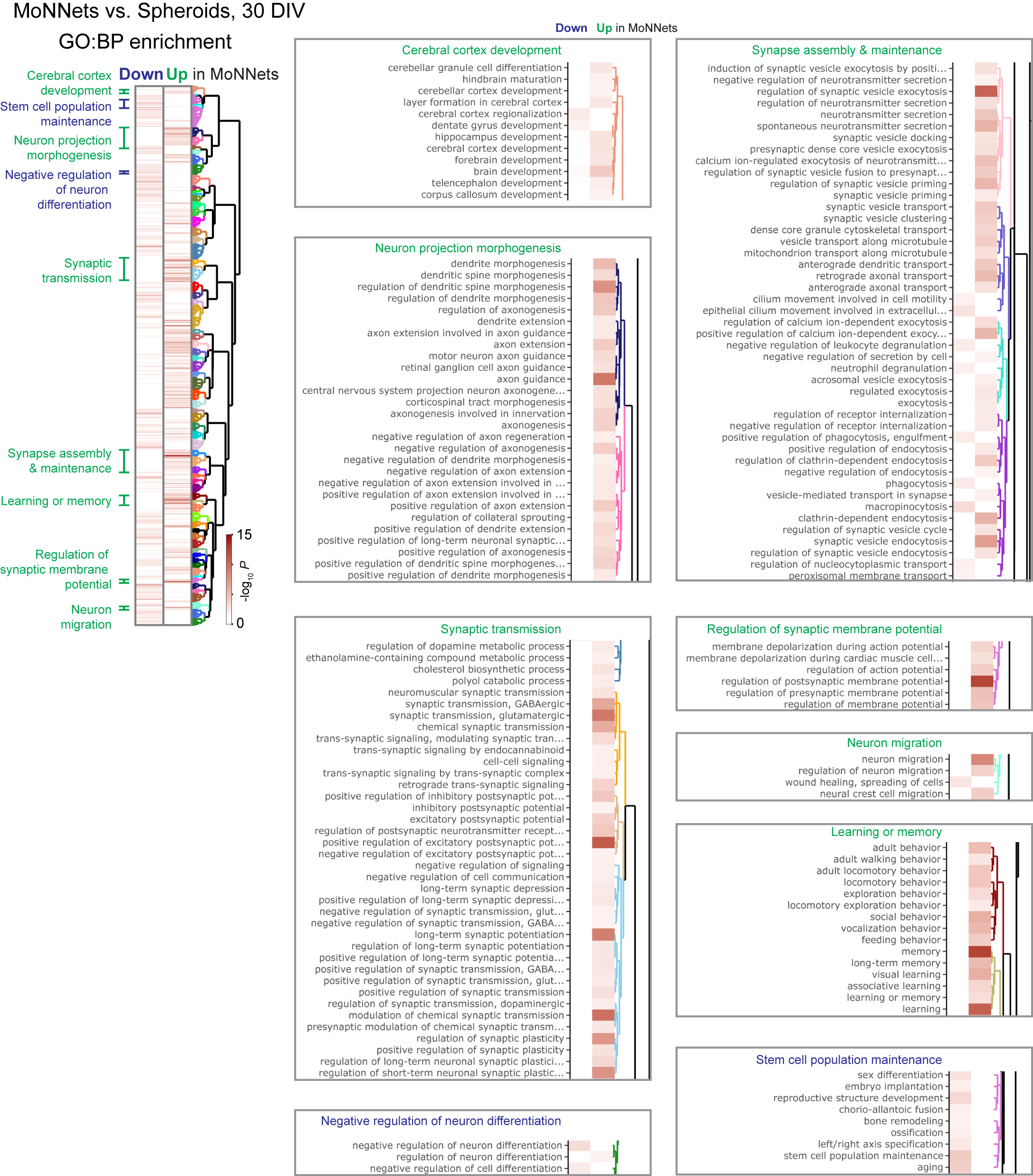


**Supplementary Fig. 10. Comparative enrichment of Gene Ontology: Biological Process terms in up (green) and down (blue) regulated genes in MoNNets, relative to spheroids.**


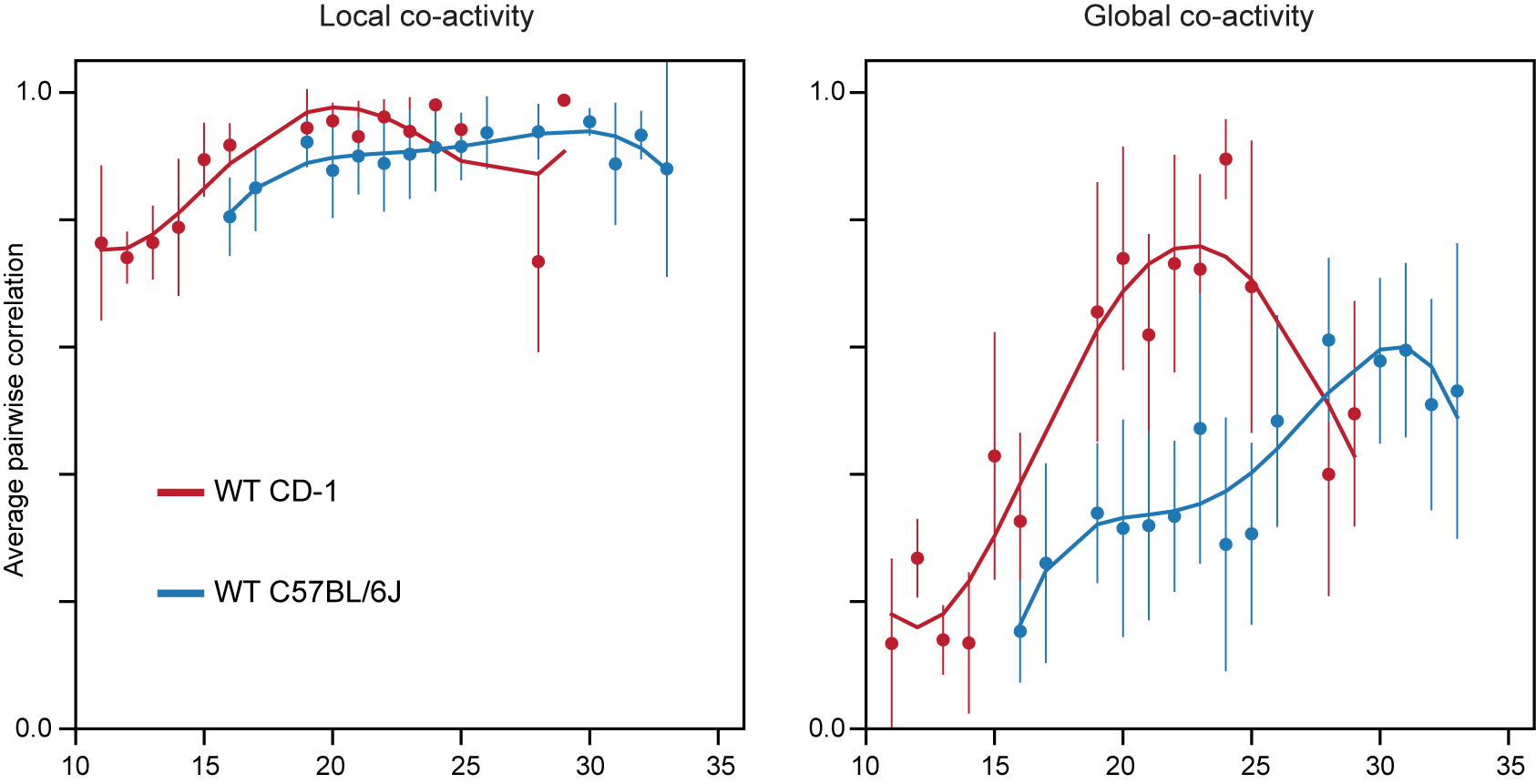


**Supplementary Fig. 11. Comparison of MoNNets derived from WT CD-1 and WT C57BL/6J.** Average pairwise correlation of MoNNets derived from WT CD-1 vs. WT C57BL/6J. Local and global average pairwise correlation of MoNNets derived from WT CD-1 (red) and C57BL/6J (blue). For WT C57BL/6J, biologically independent samples (n) across DIVs in graph as follows. n(16): 12, n(17): 6, n(19): 7, n(20): 8, n(21): 14, n(22): 13, n(23): 12, n(24): 14, n(25): 6, n(26): 7, n(28): 7, n(29): 8, n(30): 6, n(31): 9, n(32): 9, n(33): 5. For WT CD-1, biologically independent samples (n) across DIVs in graph as follows. n(11): 7, n(12): 5, n(13): 9, n(14): 27, n(15): 11, n(16): 7, n(19): 28, n(20): 33, n(21): 14, n(22): 29, n(23): 9, n(24): 10, n(25): 7, n(28): 3, n(29). For all plots, error bars are std. deviations and center points are mean values.


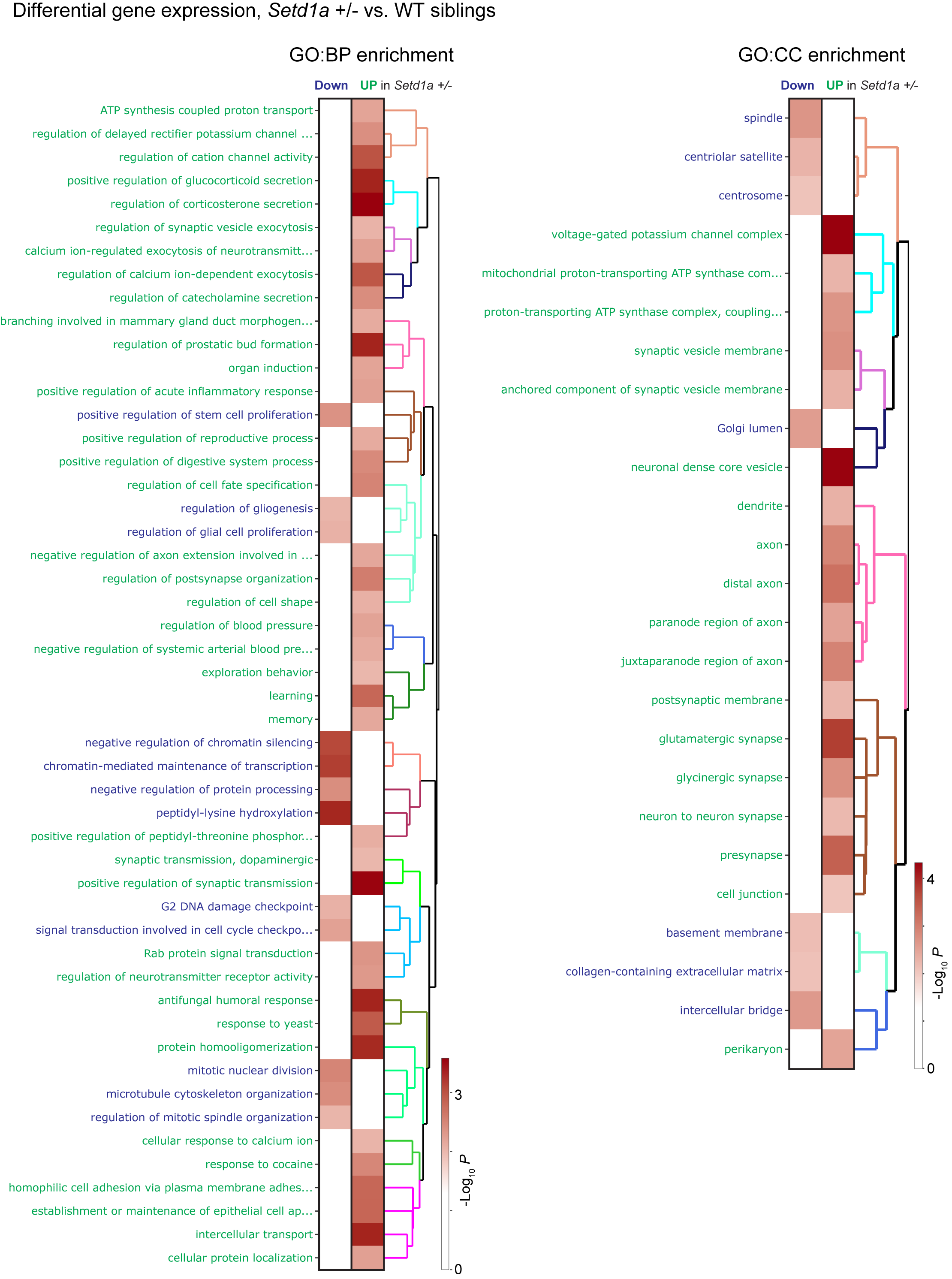


**Supplementary Fig. 12. Comparative enrichment of Gene Ontology: Biological Process and Cellular Component terms in up (green) and down (blue) regulated genes in *Setd1a* +/-, relative to WT siblings.**

**Supplementary Videos:**

**Supplementary Video 1. Representative calcium imaging data of individual spheroids and highly interconnected MoNNet.** The samples were recorded for five minutes at 30 frames per second. Only a subset of data is shown. Scale bar is 500 μm.

**Supplementary Video 2. Representative calcium imaging data of MoNNets in three phases.** The samples were recorded for five minutes at 30 frames per second. Only a subset of data is shown. Scale bar is 500 μm.

**Supplementary Video 3. Representative calcium imaging data of isolated spheroids in three phases.** The samples were recorded for five minutes at 30 frames per second. Only a subset of data is shown. Scale bar is 500 μm.

**Supplementary Video 4. Representative calcium imaging data of older MoNNet samples.** The samples were recorded for five minutes at 30 frames per second. Left: DIV 49 and Right: DIV 64. Scale bar is 500 μm.

**Supplementary Video 5. Representative calcium imaging data before and after Bicuculine [10μM] treatment.** The samples were recorded for five minutes at 30 frames per second. Only a subset of data is shown. Scale bar is 500 μm.

**Supplementary Video 6. Representative calcium imaging data of controls for the pharmacological treatments.** The samples were recorded for five minutes at 30 frames per second. Only a subset of data is shown. Scale bar is 500 μm.

**Supplementary Video 7. Confocal z-stack movie visualizing the 3D cellular architecture of MoNNet.** A Confocal z-stack visualizing DAPI (blue), GFAP (white), GCaMP6f (green) and Tuj1 (red) labels. Scale bar is 100 μm.

**Supplementary Video 8. Representative calcium imaging data from WT, *Setd1a+/-* and *Df(16)A+/-* MoNNets.** The samples were recorded for five minutes at 30 frames per second. Only a subset of data is shown. Scale bar is 500 μm.
