# Supplementary material for: An in vitro model of neuronal ensembles
Source: Nat Commun. 2022 Jun 9;13:3340. doi: 10.1038/s41467-022-31073-1 (PMC9184643; doi:10.1038/s41467-022-31073-1)
Supplement: Supplementary file 2 — Description to Additional Supplementary Information [file 41467_2022_31073_MOESM2_ESM.pdf]

Description of Additional Supplementary Files

Supplementary Video 1 |

Representative calcium imaging data of individual spheroids and highly interconnected MoNNet

Supplementary Video 2 |

Representative calcium imaging data of MoNNets in three phases

Supplementary Video 3 |

Representative calcium imaging data of isolated spheroids in three phases

Supplementary Video 4 |

Representative calcium imaging data of older MoNNet samples

Supplementary Video 5 |

Representative calcium imaging data before and after Bicuculine [10μM] treatment

Supplementary Video 6 |

Representative calcium imaging data of controls for the pharmacological treatments

Supplementary Video 7 |

Confocal z-stack movie visualizing the 3D cellular architecture of MoNNet

Supplementary Video 8 |

Representative calcium imaging data from WT, Setd1a+/- and Df(16)A+/- MoNNets
