# Supplementary material for: An in vitro model of neuronal ensembles
Source: Nat Commun. 2022 Jun 9;13:3340. doi: 10.1038/s41467-022-31073-1 (PMC9184643; doi:10.1038/s41467-022-31073-1)
Supplement: Supplementary file 11 — Reporting Summary [file 41467_2022_31073_MOESM11_ESM.pdf]

nature portfolio

Corresponding author(s): Raju Tomer

Last updated by author(s): May 25, 2022

Reporting Summary

Nature Portfolio wishes to improve the reproducibility of the work that we publish. This form provides structure for consistency and transparency in reporting. For further information on Nature Portfolio policies, see our Editorial Policies and the Editorial Policy Checklist.

Statistics

For all statistical analyses, confirm that the following items are present in the figure legend, table legend, main text, or Methods section.

n/a	Confirmed	
☐	☒	The exact sample size (*n*) for each experimental group/condition, given as a discrete number and unit of measurement
☐	☒	A statement on whether measurements were taken from distinct samples or whether the same sample was measured repeatedly
☐	☒	The statistical test(s) used AND whether they are one- or two-sided<br>*Only common tests should be described solely by name; describe more complex techniques in the Methods section.*
☒	☐	A description of all covariates tested
☐	☒	A description of any assumptions or corrections, such as tests of normality and adjustment for multiple comparisons
☐	☒	A full description of the statistical parameters including central tendency (e.g. means) or other basic estimates (e.g. regression coefficient) AND variation (e.g. standard deviation) or associated estimates of uncertainty (e.g. confidence intervals)
☐	☒	For null hypothesis testing, the test statistic (e.g. *F*, *t*, *r*) with confidence intervals, effect sizes, degrees of freedom and *P* value noted<br>*Give P values as exact values whenever suitable.*
☒	☐	For Bayesian analysis, information on the choice of priors and Markov chain Monte Carlo settings
☒	☐	For hierarchical and complex designs, identification of the appropriate level for tests and full reporting of outcomes
☒	☐	Estimates of effect sizes (e.g. Cohen's *d*, Pearson's *r*), indicating how they were calculated

Our web collection on statistics for biologists contains articles on many of the points above.

Software and code

Policy information about availability of computer code

Data collection	Micro-Manager (v1.4.22), HCImage (v4.5), Zeiss LSM700 Confocal microscope software
Data analysis	ImageJ/Fiji (v1.53c), MOCO ImageJ plugin (first and only version), Python packages Numpy (v1.190), scikit-image (v0.15), SciPy (v1.190), Caiman (1.6.3), NetworkX (2.4) and MATLAB (R2020b).

For manuscripts utilizing custom algorithms or software that are central to the research but not yet described in published literature, software must be made available to editors and reviewers. We strongly encourage code deposition in a community repository (e.g. GitHub). See the Nature Portfolio guidelines for submitting code & software for further information.

Data

Policy information about availability of data

All manuscripts must include a data availability statement. This statement should provide the following information, where applicable:

- Accession codes, unique identifiers, or web links for publicly available datasets
- A description of any restrictions on data availability
- For clinical datasets or third party data, please ensure that the statement adheres to our policy

All data are available from authors upon request. Bulk RNA sequencing data are available under BioProject accession code PRJNA832747. Publicly available Mef2 data used is available under accession code GEO: GSE123652 (Mukai, J. et al. Neuron, doi:10.1016/j.neuron.2019.09.014 (2019)). Source data are provided as a Source Data file.

Field-specific reporting

Please select the one below that is the best fit for your research. If you are not sure, read the appropriate sections before making your selection.

☒ Life sciences ☐ Behavioural & social sciences ☐ Ecological, evolutionary & environmental sciences

For a reference copy of the document with all sections, see nature.com/documents/nr-reporting-summary-flat.pdf

Life sciences study design

All studies must disclose on these points even when the disclosure is negative.

Sample size	Large samples sizes (e.g. 282 independent samples for the analysis shown in Figure 1) were used for all experiments, as detailed in the text and figure legends, to minimize the effects of sample-to-sample variations and heterogeneity, which was not possible to estimate beforehand. No statistical method was used to predetermine sample sizes.
Data exclusions	Pre-established criteria was used to exclude MoNNet samples showing no neuronal activity irrespective of sample group.
Replication	As described for all data figures, large number of biological and technical replicates were used and are detailed in the corresponding figures and text.
Randomization	Animals were grouped based on their genotype, which was confirmed by tail PCR for all individual animals.
Blinding	As the animals were to be grouped based on their genotype (determined by tail PCR), no blinding was possible for this.

Reporting for specific materials, systems and methods

We require information from authors about some types of materials, experimental systems and methods used in many studies. Here, indicate whether each material, system or method listed is relevant to your study. If you are not sure if a list item applies to your research, read the appropriate section before selecting a response.

Materials & experimental systems

n/a	Involved in the study	
☐	☒	Antibodies
☒	☐	Eukaryotic cell lines
☒	☐	Palaeontology and archaeology
☐	☒	Animals and other organisms
☒	☐	Human research participants
☒	☐	Clinical data
☒	☐	Dual use research of concern

Methods

n/a	Involved in the study	
☒	☐	ChIP-seq
☒	☐	Flow cytometry
☒	☐	MRI-based neuroimaging

Antibodies

Antibodies used	Rabbit anti-Glutaminase (1:500, Cat# Gltn-Rb-Af340, Frontier Institute Co. LTD.), mouse anti-NeuN, clone A60 (1:50, Cat# MAB377, Millipore), mouse anti-GAD65 (1:1000, GAD-6, DSHB), rabbit anti-GFAP (1:500, Cat# Z0334, Dako), rat anti-phospho-Histone H3 (1:200, Cat# h9908, Sigma), mouse anti-TUJ1 (1:200, Cat# 801201, BioLegend) and rabbit anti-Caspase3(1:500, Cat# 559565, BD Pharmingen). Alexa Fluor 568-, and 647- conjugated secondary antibodies (goat anti rabbit 647 cat# A21245, goat anti Rabbit 568 cat# A11011, goat anti Mouse 647 cat# A21235, goat anti Mouse IgG2a 647 cat#A21241, Invitrogen) were used at 1:500 dilutions.
Validation	As noted above, standard widely used commercial antibodies were used for stainings. We also performed appropriate control experiments with secondary antibody only and also inspected of would-be-expected staining patterns (known from literature).

Animals and other organisms

Policy information about studies involving animals; ARRIVE guidelines recommended for reporting animal research

Laboratory animals	WT mice strains CD-1 and C57BL/6J, and mutant Df(16)A+/– mice (RRID: MGI_3802827) and Setd1a+/- mice (EMMA) were used in this study.
Wild animals	No Wild animals were used in this study.
Field-collected samples	No field-collected samples or animals were used.
Ethics oversight	Institutional Animal Care and Use Committees(IACUC) of Columbia University.

Note that full information on the approval of the study protocol must also be provided in the manuscript.
